# Phenotypic variation and genetic architecture of seedling salt tolerance in Xinjiang wheat revealed by GWAS and resequencing

**DOI:** 10.3389/fpls.2026.1847734

**Published:** 2026-07-02

**Authors:** Guiqiang Fan, Jia-nan Huang, Hong-jin Wang, Yindeng Ding, Yonghong Gao, Tulafu Tuhexun, Uzair Ullah, Zikun Wang, Lubna Khan, Abdullah Shalmani, Hui Fang, Tianrong Huang

**Affiliations:** 1 Institute of Crop Research, Academy of Agricultural Sciences of Xinjiang Uyghur Autonomous Region, Urumqi, Xinjiang Uyghur Autonomous Region, China; 2 School of Materials Science and Engineering, Jingdezhen Ceramic University, Jingdezhen, Jiangxi, China; 3 Xinjiang Jiufenghe Seed Industry Co., Ltd., Urumqi, Xinjiang Uyghur Autonomous Region, China; 4 National Key Laboratory of Crop Improvement for Stress Tolerance and Production, College of Life Sciences, Northwest A&F University, Yangling, Shaanxi, China; 5 School of Biological Science, Shaanxi University of Technology, Hanzhong, China

**Keywords:** resequencing, salt stress, salt tolerance index, seedling traits, wheat

## Abstract

**Introduction:**

Salt stress is a major constraint on wheat production, particularly in arid and semi-arid regions such as Xinjiang, China. Understanding the phenotypic variation and genetic basis of salt tolerance at the seedling stage is essential for improving wheat breeding efficiency.

**Methods:**

A total of 134 wheat accessions from Xinjiang were evaluated under control (0 mM Na₂SO₄) and salt stress (120 mM Na₂SO₄) conditions. Seedling traits including plant height (PH), root length (RL), and germination rate (GR) were measured. Salt tolerance indices (STIs) were calculated to quantify stress responses. Genome-wide association studies (GWAS) were performed using resequencing-derived SNP data and linear mixed models (LMMs). Transcriptome data from salt-tolerant and salt-sensitive genotypes were integrated to prioritize candidate genes.

**Results:**

Significant phenotypic variation was observed among accessions under salt stress. GWAS identified multiple SNPs significantly associated with salt tolerance traits, which were clustered into 18 robust QTL intervals, including major hotspots on chromosomes 2A and 2B. Transcriptome analysis further supported the identification of candidate genes showing differential expression between tolerant and sensitive genotypes.

**Discussion:**

The integration of GWAS and transcriptomic data provided a more comprehensive understanding of the genetic architecture underlying seedling-stage salt tolerance in wheat. The identified QTLs and candidate genes offer valuable resources for functional validation and molecular breeding of salt-tolerant wheat varieties.

## 1 Introduction

Soil salinization is a major constraint on global agricultural production, especially in arid and semi-arid regions (Rengasamy, 2006). High evaporation, low precipitation, and imbalances in irrigation and drainage promote the accumulation of salts in the topsoil, leading to a decline soil productivity and an increasing extent of salinized land (Tarolli et al., 2024). Salt stress adversely effects crop growth throughout entire life cycle, but its impact is especially critical during germination and early seedling establishment. Successful seed germination depends on adequate and stable water uptake; however, elevated salinity reduces external water potential, including osmotic stress that restricts water absorption, delays germination, or even causes complete failure (Zhu, 2001; Manono, 2025; Gul et al., 2013). In addition, the influx of Na^+^ and SO_4_²^-^ ions disrupts cellular ion homeostasis and inhibits key enzymatic activities, resulting in ion toxicity (Tester and Davenport, 2003; Reginato et al., 2021). Prolonged salt stress further triggers excessive accumulation of reactive oxygen species (ROS), leading to membrane lipid peroxidation and cellular damage. These processes ultimately inhibit cell elongation and division, thereby reducing root growth, seedling height, and overall seedling vigor (Arif et al., 2020; Dang et al., 2025). These multiple effects ultimately lead to reduced emergence rate, poor seedling establishment, and a reduction in the number of effective ears, thus profoundly impacting yield.

Wheat (Triticum aestivum L.) is one of the most important staple crops worldwide and play a fundamental role in China’s food security (Shiferaw et al., 2013; Li et al., 2019). With increasing pressure on arable land resources and the expansion of cultivation into marginal environments, improving wheat production in saline-alkali soils and enhancing salt tolerance have become critical breeding objective (Lei et al., 2025; EL Sabagh et al., 2021; Traye et al., 2025). Salt tolerance in wheat is a complex quantitative trait governed by coordinated regulation of multiple physiological processes and molecular networks. Salt tolerance in wheat is a complex quantitative trait involving ion homeostasis, osmotic adjustment, antioxidant defense, and stress-signaling pathways, including hormone- and calcium-mediated regulatory networks. In addition, the hexaploidy nature of the wheat genome introduce further complexity, as gene redundancy, allelic variation, and sub-genome-specific expression differentiation complicate the genetic dissection of salt tolerance and hinder the identification favorable alleles associated with stress adaptation (Appels et al., 2018; Glombik et al., 2025; Oyiga et al., 2018; Zheng et al., 2022).

Evaluation and screening of salt tolerance at the seedling stage are crucial steps in salt-tolerant breeding. Seedling traits such as plant height, root length, and germination rate can be measured rapidly and consistently, making them suitable for large-scale phenotypic screening of germplasm resources (Khan et al., 2022). In particular, the root system plays are a central role in salt stress perception and ion uptake; therefore, root-related traits such as root length are sensitive indicators of stress response. Germination rate, in turn, is closely associated with population establishment efficiency and early seedling vigor under stress conditions (Cui et al., 2025; Finch-Savage and Bassel, 2016). Although single traits provide valuable insights, salt tolerance is inherently a multidimensional trait resulting from integration of multiple physiological processes. Therefore, the development of composite evaluation indices is of practical importance (Li et al., 2021). Salt tolerance indices (STIs) are widely used to evaluate stress tolerance because they normalize trait performance under stress relative to control conditions and facilitate comparisons among genotypes (Li et al., 2021; Chaurasia, 2024).

At the genetic analysis level, traditional QTL linkage mapping relies on biparental populations and limited recombination events, resulting in relatively large confidence interval that restrict its directly application in fine mapping and candidate gene identification (Xu et al., 2017; Singh et al., 2025). In contrast, advances in high-throughput sequencing and genomics have enabled genome-wide association studies (GWAS) based on natural or diverse populations to become a powerful approach for dissecting the genetic basis of complex traits (Rafalski, 2010). GWAS exploits historical recombination within populations to achieve higher mapping resolution and, when combined with high-density SNP markers, enables the identification of traits associated loci across the entire genome (Mackay and Powell, 2007; Yu et al., 2006). In wheat, multiple loci associated with salt tolerance have been identified at different developmental stages and under different stress conditions, highlighting the polygenic nature of this trait (Song et al., 2025; Hu et al., 2021). However, GWAS is susceptible to confounding effects arising from population structure and relatedness, which can lead to false positives associations (Pritchard and Rosenberg, 1999). To address this, linear mixed models (LMMs) are widely employed, incorporating kinship matrix as random effects and population structure (e.g., principal component) as fixed-effect covariates to improve the accuracy and robustness of association signals (Kang et al., 2008; Patterson et al., 2006; Bradbury et al., 2007). Nevertheless, given salt tolerance is usually influenced by numerous loci with small effects and influenced by environmental interactions, individual significant SNPs often provide limited biological insight (Boyle et al., 2017; Korte and Farlow, 2013; Walsche et al., 2025). Therefore, clustering associated SNPs into robust QTL intervals and integrating these regions with functional annotation and gene expression evidence are essential strategies for prioritizing candidate genes and enhancing the biological interpretability of GWAS results (Gou et al., 2026; Kaňovská et al., 2024).

Transcriptomics analysis provides important complementary evidence for candidate gene identification (Wu et al., 2025). RNA sequencing (RNA-seq) captures gene expression dynamics across different genotypes and treatment conditions, thereby serving as a crucial link between genotype and phenotype. In particular, comparative analysis between salt-tolerant and salt-sensitive genotypes under control and stress conditions enables the identification of genes exhibiting differential stress-responsive expression patterns. Such comparative frameworks facilitate the prioritization of candidate genes potentially involved in salt tolerance, there materials, thereby narrowing the candidate pool and improving the efficiency of downstream validation (Wu et al., 2025; Love et al., 2014; Cheng et al., 2024). Integrating GWAS/QTL mapping with transcriptomic data further strengthens candidate gene selection by combining genetic association signals with expression evidence. This multi-layered approach enhances the biological relevance and interpretability of candidate genes and provides a more robust basis for subsequent functional validation.

Xinjiang, located in the arid northwest region of China, is characterized by extensive saline-alkali soils and represent one of the country’s major wheat production and breeding regions (Zhang et al., 2024; Zhang et al., 2025; Gao et al., 2025). Wheat germplasm resources from Xinjiang include landraces, introduced accessions, and modern cultivars, which have accumulated substantial genetic diversity and environmental adaptability through long-term ecological adaptation and artificial selection (Lopes et al., 2015; Ma et al., 2020). Compared with populations composed solely modern cultivars, Xinjiang local germplasm often retains a greater abundance of rare variants and adaptive alleles, making it a valuable resource for identifying new loci and genes associated with salt tolerance (Ma et al., 2020; Dai et al., 2020). Despite these advantages, systematic studies integrating seedling-stage phenotypic evaluations, high-resolution GWAS based on resequencing-derived SNP data, and transcriptomic-assisted candidate genes prioritization in Xinjiang wheat remain limited. This lack of integrative analysis has constrained the discovery of salt tolerance-related genetic loci and hindered the development and utilization of molecular markers for salt-tolerant wheat breeding (Ma et al., 2020; Huang et al., 2026; Javid et al., 2022).

Based on these considerations, the present study evaluated 134 wheat accessions from Xinjiang under control (0 mM Na_2_SO_4_) and salt stress (120 mM Na_2_SO_4_) conditions by measuring three seedling-stage traits: plant height (PH), root length (RL), and germination rate (GR). Salt tolerance indices (STIs) and composite index (STI_MEAN) were calculated to systematically evaluate phenotypic variation, trait correlation, and differentiation in salt tolerance among accessions. To investigate the genetic basis of seedling-stage salt tolerance, genome-wide association studies (GWAS) were conducted using resequencing-derived genome-wide SNP data and linear mixed models (LMMs), followed by integration of significant loci into robust QTL intervals to identify key genomic regions related with salt tolerance. In addition, transcriptome expression profiles from salt-tolerant and salt-sensitive genotypes under control and salt stress conditions were integrated with GWAS/QTL results to prioritize candidate genes showing differential salt responsive expression patterns. Recent genome-wide association studies (GWAS) have identified numerous loci associated with salt tolerance in wheat, highlighting the complex genetic architecture of salinity adaptation. However, most previous studies have focused primarily on NaCl stress, whereas the genetic basis of Na_2_SO_4_ tolerance remains less explored. Therefore, investigating Na_2_SO_4_ tolerance in Xinjiang wheat germplasm may provide additional insights into the genetic mechanisms underlying salt adaptation. The specific objectives of this study were to: (1) characterize phenotypic variation in seedling-stage salt tolerance and identify superior germplasm resources in Xinjiang wheat; (2) detect key SNPs and robust QTL intervals associated with salt tolerance-related traits; and (3) prioritize candidate genes within QTL regions based on transcriptomic evidence, thereby providing a foundation for future functional validation and molecular marker development for salt-tolerant wheat breeding.

## 2 Materials and methods

### 2.1 Test materials

A total of 134 wheat (*Triticum aestivum L*.) accessions originating from Xinjiang were used in this study. All accessions were modern cultivars or breeding lines and were uniformly designated as xj1–xj134. Seed used for phenotypic evaluation were harvested in the same growing season and maintained under consistent storage conditions prior to experimentation to minimize variation associated with seed age and storage environment. All accessions were subjected to identical salt stress treatment, phenotypic measurements, and subsequent genetic analysis under standardized experimental.

### 2.2 Salt stress treatment and germination culture

Salt treatment was performed using sodium sulfate (Na_2_SO_4_) solution to stimulate sulfate-dominated saline conditions commonly observed in Xinjiang saline-alkali soils (Wang et al., 2025). Two treatment conditions were established: distilled water as the control (CK, 0 mM Na_2_SO_4_) and 120 mM Na_2_SO_4_ as the salt stress treatment. The Na_2_SO_4_ solution was prepared by dissolving appropriate amount of sodium sulfate in distilled water and mixing thoroughly before use. Germination tests were conducted in petri dishes lined with two layers of filter paper. For each accession and treatments, 100 seeds were evenly distributed in each dish, and an equal volume of either distilled water (CK) or Na_2_SO_4_ solution was added to maintain consistent moisture conditions throughout the experiment. Three independent biological replicates were conducted for each treatment. All germination and seedling growth assays were carried out in a controlled growth chamber at 25 ± 1°C under a 16 h light/8 h dark photoperiod, with a light intensity of approximately 250 µmol m^-2^ s^-1^ and relative humidity maintained at 65-70%. A seed was considered germinated when radical protruded at least 2 mm. Phenotypic measurements were recorded 7 days after germination, and all accessions were evaluated at the same developmental stage to minimize variation caused by developmental heterogeneity.

### 2.3 Phenotypic determination

Seedling-stage phenotypic traits, including plant height (PH), root length (RL), and germination rate (GR), were evaluated under control (CK, 0 mM Na_2_SO_4_) and salt stress (120 mM Na_2_SO_4_) conditions. Germination rate was calculated as the percentage of seeds exhibiting radicle protrusion of at least 2 mm relative to the total number of seeds per dish. To minimize the influence of false germination under salt stress, seeds showing only minimal radicle emergence without subsequent seedling development were excluded from GR calculation. Plant height (PH) was measured as the distance from the seed base to the tip of the shoot, while root length (RL) was measured as the length of the primary root. Phenotypic measurements were recorded 7 days after germination under identical developmental conditions for all accessions and treatments. For each accession, measurements were obtained from three independent biological replicates, and mean values were used for subsequent statistical analyses.

### 2.4 Salt tolerance index and relative response calculation

To quantify the ability of each wheat accession to maintain growth performance under salt stress, the salt tolerance indices (STIs) were calculated based on phenotypic values obtained under control (CK, distilled water) and salt stress conditions using the following formula:


$$STI=\frac{Trait_{Na2SO4}}{Trait_{CK}}$$

Where Trait_Na_2_SO_4_ represents the phenotypic value measured under 120 mM Na_2_SO_4_ treatment, and Trait_CK represents the corresponding phenotypic value under control conditions. Separate STIs were calculated for plant (STI_PH), root length (STI_RL), and germination rate (STI_GR). To provide an integrated evaluation of seedling-stage salt tolerance, a composite salt tolerance index (STI_MEAN) was calculated as the arithmetic mean of the three individual indices:


$$STI\_MEAN=\frac{STI\_PH+STI\_RL+STI\_GR}{3}$$

Because the three evaluated traits represent distinct but complementary aspects of seedling performance under salt stress, equal weighting was applied to avoid overemphasizing any single phenotypic component. However, we acknowledge that differences in that trait distribution and variance may influence the contribution of individual indices to the composite score, and this limitation should be considered when interpreting STI_MEAN. In addition, the relative response (RR, %) of each trait under salt stress was calculated as:


$$RR(\%)=\frac{Trait_{Na2SO4}-\;Trait_{CK}}{Trait_{CK}}\;\times 100$$

Where negative RR values indicate growth inhibition under salt stress relative to the control condition.

### 2.5 Transcriptome sampling, library construction, sequencing, and expression level analysis

Root tissues were collected 7 days after germination under control conditions (CK, distilled water) and 120 mM Na_2_SO_4_ stress. Based on the phenotypic evaluation and the composite salt tolerance index (STI_MEAN), two salt-tolerant accessions (xj1 and xj2) and two salt-sensitive accessions (xj6 and xj7) were selected for comparative transcriptome analysis. For each accession and treatment, three independent biological replicates were collected, resulting in a total of 24 samples (4 accessions × 2 treatments × 3 biological replicates). Total RNA was extracted from root tissues using TRIzol Reagent (Invitrogen, USA), and genomic DNA was removed by DNase digestion before library preparation. RNA integrity and quality were assessed before library construction. Messenger RNA was enriched using oligo(dT) magnetic beads and fragmented into short fragments. First-strand and second-strand cDNA were synthesized, followed by end repair, A-tailing, adapter ligation, size selection, and PCR amplification. Library quality and insert-fragment size were evaluated using an Agilent 2100 Bioanalyzer.

Strand-specific, reverse-stranded mRNA-seq libraries were sequenced using a paired-end strategy on the BGI DNBSEQ-T7 platform. On average, approximately 6.59 Gb of clean data were generated per sample. Raw reads were quality-filtered using fastp v0.20.1 with the parameter --length required 50, meaning that reads shorter than 50 bp after trimming were discarded. Adapter sequences, low-quality reads, and low-quality bases were removed during quality control. Clean reads were aligned to the wheat reference genome IWGSC CS RefSeq v2.1 using HISAT2 v2.1.0 with the parameters --rna-strandness rf --fr. Gene-level read counts were generated using htseq-count v0.11.2 with the parameter -s reverse. FPKM values were calculated to describe gene-expression abundance, whereas raw gene-count values were used for differential-expression analysis.

Differentially expressed genes were identified using DESeq2 v1.22.2. Genes with q-value < 0.05 and |log_2_ fold change| > 1 were considered differentially expressed. To identify genes showing contrasting responses between tolerant and sensitive accessions under Na_2_SO_4_ stress, a tolerance group × treatment interaction model was applied using the design formula ~ tolerance group + treatment + tolerance group: treatment. Candidate genes within high-confidence QTL intervals were prioritized by integrating genomic position, expression abundance, fold-change magnitude, interaction significance, and functional annotation.

### 2.6 Whole-genome resequencing and reference genome

Whole-genome resequencing was performed for 134 Xinjiang wheat accessions using the BGI sequencing platform, with an average sequencing depth of approximately 10× per accession. Raw reads were quality-filtered using fastp v0.20.1 to remove adapter sequences, reads containing excessive ambiguous bases, and low-quality reads. The resulting high-quality clean reads were aligned to the Chinese Spring wheat reference genome IWGSC CS RefSeq v2.1 (NCBI RefSeq assembly: GCF_018294505.1) using BWA-MEM v0.7.17 with default parameters. Alignment files were sorted and indexed using SAMtools v1.11, and duplicate reads were marked using Picard tools before variant detection.

All downstream genomic analyses, including variant calling, SNP filtering, population-structure analysis, kinship estimation, LD-decay analysis, GWAS, and candidate-gene annotation, were performed using the same reference-genome and annotation version to ensure consistency across datasets.

### 2.7 Variant calling, quality control, and missing-data handling

SNPs and InDels were identified using the Genome Analysis Toolkit (GATK v4.1.8.0). GATK HaplotypeCaller was applied in GVCF mode for individual-level variant calling, followed by joint genotyping using GenotypeGVCFs to generate a population-level VCF file. Raw variants were subjected to hard filtering using SnpSift Filter. For SNPs, variants were retained when QD > 2.0, MQ > 40.0, FS < 60.0, SOR < 3.0, MQRankSum > −12.5, and ReadPosRankSum > −8.0. For InDels, variants were retained when QD > 2.0, FS < 200.0, and SOR < 10.0. Only high-quality biallelic SNPs were used for subsequent population-genetic and association analyses.

Before population-structure analysis, kinship estimation, LD-decay analysis, and GWAS, SNPs with a missing genotype rate greater than 10% (GENO > 0.1) were excluded, and variants with a minor allele frequency below 0.05 (MAF < 0.05) were removed. Genotype imputation was not performed; therefore, no imputation software or external reference panel was used. Variant annotation and summary statistics were generated using snpEff v5.1d and VCFtools v0.1.17, respectively.

### 2.8 Population structure and kinship

Population structure analysis was performed using ADMIXTURE software based on the filtered genome-wide SNP dataset. The number of ancestral populations (*K*) was evaluated from to 2 to 10, and optimal K value was determined using 10-fold cross-validation CV). Based on the minimum cross-validation error, the optimal population structure was identified at *K* = 3. The account for potential confounding effects caused by population stratification and genetic relatedness in GWAS, a genomic relationship matrix (GRM) was constructed using GCTA software based on the filtered genome-wide SNP markers (Yang et al., 2011). The GRM was subsequently incorporated into the Linear Mixed Model (LMM) for GWAS analysis to improve the reliability of marker-trait association and reduce false-positive signals arising from population structure and kinship effects.

### 2.9 Genome-wide association analysis and QTL integration

Genome-wide association analysis (GWAS) was performed using multi-trait phenotype datasets, including STI_PH, STI_RL, STI_GR, and STI_MEAN. Initial association scans were performed using generalized linear model (GLM) implemented in PLINK, while the primary association analysis was conducted using GCTA under a Linear Mixed Model (LMM) framework with genomic relationship matrix (GRM) correction to control for population structure and kinship effects (Yang et al., 2011; Purcell et al., 2007; Yang et al., 2014). The GWAS workflow included VCF to PLINK format conversion, SNP quality filtering based on missing genotype rate (GENO > 0.1) and minor allele frequency (MAF < 0.05) (Niu et al., 2023); and GRM construction using the GCTA “--make-grm” function followed by mixed-model association analysis using “—mlma” function (Yang et al., 2014). Chromosome information was standardized according to the 21 chromosomes of the hexploid wheat genome (AABBDD). A significance threshold of P < 1×10^-5^ was used for visualization and extraction of significant associations signals. Manhattan plots, quantile-quantile (QQ) plots, and summary tables of significant SNPs were generated using R scripts. Significant SNPs were clustered into QTL intervals based on physical proximity on the reference genome, candidate genes within QTL regions were prioritized by integrating gene annotations and transcriptome expression data from salt-tolerant and salt-sensitive accessions.

### 2.10 PCA analysis

Principal component analysis (PCA) was performed to evaluate the overall similarity among RNA-seq samples and to assess biological reproducibility. The normalized gene expression matrix of the 24 root RNA-seq samples was transformed as log2(expression + 1). Genes with non-zero expression were retained, and the 500 genes with the highest variance across samples were used for PCA, following a commonly used transcriptome quality-control strategy for sample-level clustering. PCA was conducted by singular value decomposition. In the PCA plot, samples were annotated according to eight material/treatment groups: Material-1 CK, Material-1–120 mM Na2SO4, Material-2 CK, Material-2–120 mM Na2SO4, Material-6 CK, Material-6–120 mM Na2SO4, Material-7 CK, and Material-7–120 mM Na2SO4.

### 2.11 Software and environment

The main software used for resequencing data analysis included fastp v0.20.1 for read-quality filtering, BWA-MEM v0.7.17 for reference-genome alignment, SAMtools v1.11 for alignment-file processing, Picard tools for duplicate-read marking, GATK v4.1.8.0 for variant calling and joint genotyping, SnpSift Filter for variant-quality filtering, snpEff v5.1d for variant annotation, and VCFtools v0.1.17 for variant statistics. PLINK was used for genotype-format conversion and SNP filtering, GCTA was used for genomic relationship matrix construction and Linear Mixed Model-based GWAS, ADMIXTURE was used for population-structure analysis, and R was used for statistical analysis and visualization. All bioinformatics analyses were performed in a Linux environment.

## 3 Results

### 3.1 Na_2_SO_4_ stress significantly inhibited seedling height, root length, and germination rate

**Figure 1:**
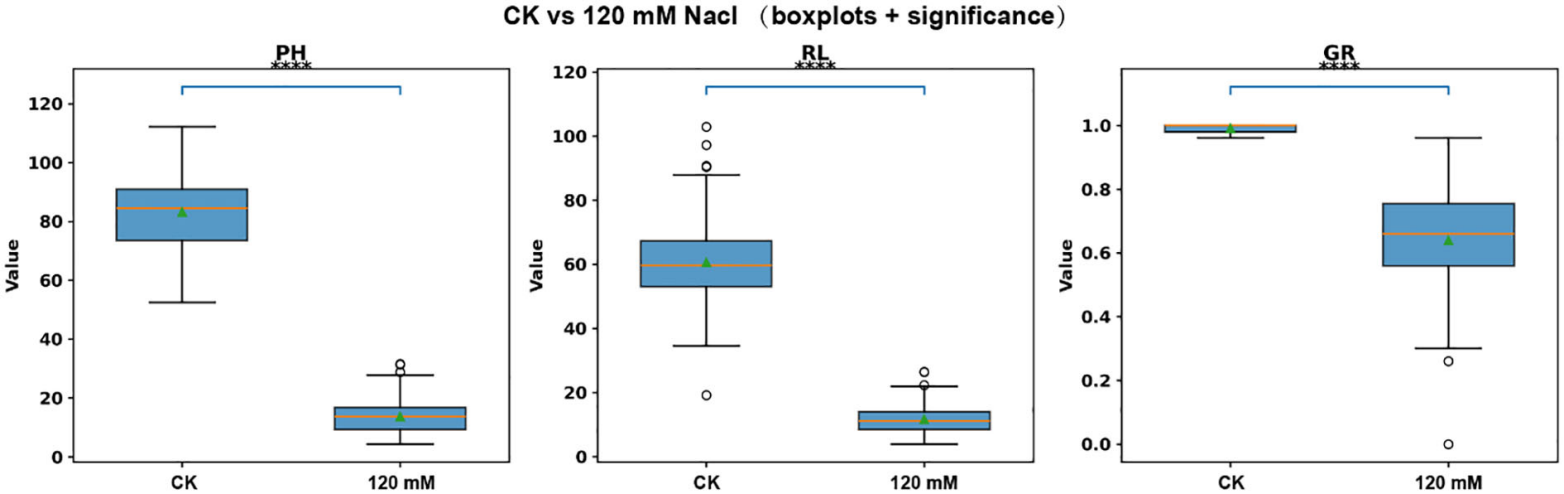
Phenotypic differences in plant height (PH), root length (RL), and germination rate (GR) of 134 Xinjiang wheat accession under control (CK, 0 mM Na_2_SO_4_) and Na_2_SO_4_ stress (120 mM Na_2_SO_4_) conditions. Box plots show the median and interquartile range, whiskers indicated the data range, and points represent individual accessions or outliers. Asterisks above brackets indicate significant differences between CK and Na_2_SO_4_ stress based on paired tests.

Under the control condition (CK, 0 mM Na_2_SO_4_), the mean plant height (PH), root length (RL), and germination rate (GR) of the 134 Xinjiang wheat accessions were 83.25, 60.62, and 0.992, respectively. Under 120 mM Na_2_SO_4_ stress, PH, RL, and GR decreased to 13.72, 11.68, and 0.645, respectively, indicating that Na_2_SO_4_ stress significantly inhibited seedling growth and germination (**Figure 1**). Trait variation increased significantly under salt stress, with coefficients of variation (CVs) of 40.32% for PH and 37.26% for RL, suggesting substantial differences in salt-stress responses among accessions. Paired difference tests further confirmed that the differences between CK and 120 mM Na_2_SO_4_ were highly significant for all three traits: PH (t-test P = 6.32 × 10^-^¹^0^³; Wilcoxon P = 9.80 × 10^-^²^4^), RL (t-test P = 4.08 × 10^-^^86^; Wilcoxon P = 9.80 × 10^-^²^4^), and GR (t-test P = 6.37 × 10^-^^55^; Wilcoxon P = 9.49 × 10^-^²^4^). Relative responses analysis showed that Na_2_SO_4_-stress had a stronger inhibitory effect on seedling growth traits than on germination rate. The mean relative responses were -83.35% for PH, -80.43% for RL, and -35.51% for GR.

### 3.2 Phenotypic distribution and normality assessment

To evaluate phenotypic distribution patterns and the suitability of subsequent statistical analyses, distribution fitting and normality tests were performed for 10 traits, including PH, RL, and GR under control and 120 mM Na_2_SO_4_ stress conditions, as well as the corresponding salt tolerance index trait. Overall, the PH_CK and RL_CK showed distributions relatively close to normal, whereas GR-related traits showed skewed distributions and clear ceiling effect because GR values were close to the upper limit of 0–1 under control conditions. PH and RL under Na_2_SO_4_ stress also showed some deviation from normality, indicating substantial phenotypic differentiation among accessions under stress condition (**Supplementary Figure S8**). The quantile-quantile plots further confirmed that several traits deviated from the theoretical normal distribution line (**Supplementary Figure S5**). Therefore, both parametric and non-parametric paired tests were used when comparing control and stress treatments and linear mixed model-based GWAS was applied to reduce confounding effects from population structure and relatedness.

### 3.3 Salt tolerance index characterizes variation in Na_2_SO_4_ tolerance and identifies extreme accessions

Salt tolerance indices (STI) were calculated as the ratio of trait performance under 120 mM Na_2_SO_4_ stress to the corresponding performance under control conditions. The mean values of the three single-trait indices were 0.166 for STI_PH, 0.196 for STI_RL, and 0.645 for STI_GR, with ranges of 0.050-0.070-0.499, and 0-0.960, respectively. The composite index STI_MEAN had a mean value of 0.36 and ranged from 0.069 to 0.473, indicating substantial variation in the ability of different accessions to maintain seedling growth and germination under Na_2_SO_4_ stress (**Supplementary Figure 7**). Based on STI_MEAN, the accessions were ranked to identify extreme tolerant and sensitive materials. The Top 10 and Bottom 10 accessions, together with their key phenotypic traits and STI values, are summarized in **Supplementary Table 1**. In general, the top-ranked accessions maintained higher PH and RL retention and showed smaller reduction in GR under Na_2_SO_4_ stress, whereas the bottom-ranked accessions showed severe reduction in pH, RL and GR, indicating high sensitivity at the seedling stage.

Because PH, RL, and GR differed in distribution and coefficient of variation, we further evaluated the robustness of the equal-weighted STI_MEAN using alternative composite indices. STI_MEAN was highly correlated with both the Z-score-based index and the PCA-based index, with Pearson correlation coefficients of 0.960 and 0.956, respectively. The top 20% tolerant accessions also showed high overlap among the three ranking methods. These results indicate that the equal-weighted STI_MEAN provides a robust and interpretable summary of seedling-stage Na_2_SO_4_ tolerance in this population, although trait-specific contributions should still be considered when interpreting individual accessions (**Supplementary Figure S11**).

### 3.4 Trait correlation and pairwise relationships reveal coordinated and trait-specific responses to Na_2_SO_4_ stress

**Figure 2:**
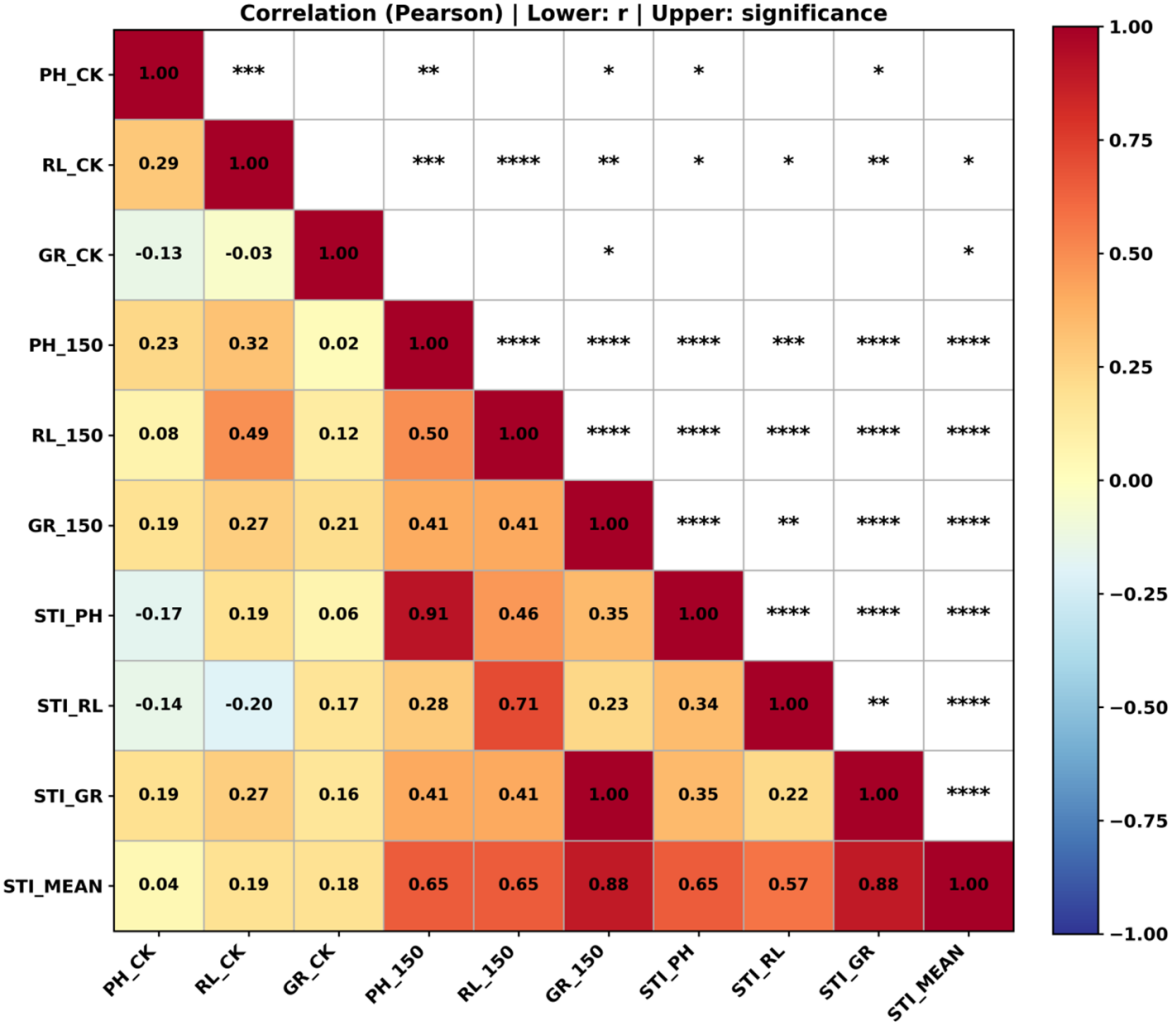
Pearson correlation heatmap of 10 seedling-stage traits in 134 Xinjiang wheat accessions. The traits include plant height, root length, and germination rate under control conditions (PH_CK, RL_CK, GR_CK), and 120 mM Na_2_SO_4_ stress conditions (PH_120, RL_120, GR_120, STI_PH, STI_RL, STI_GR), and the composite index STI_MEAN. The lower triangle shows the Pearson correlation coefficient, and the upper triangle shows significance level. The color scale indicates the magnitude and direction of correlations, and hierarchical clustering shows the correlation structure between traits. *: P < 0.05; **: P < 0.01; ***: P < 0.001; ****: P < 0.0001.

The Pearson correlation analysis showed strong consistency between each salt tolerance index and its corresponding trait under 120 mM Na_2_SO_4_ stress. The correlation coefficient between STI_PH and PH under stress was approximately 0.91, while STI_RL was positively correlated with RL under stress (r = 0.71). STI_GR and GR under stress were almost completely correlated (r = 1.00), mainly because GR under control conditions was close to 1.0 for most accessions (**Figure 2**). These results indicate that the STI values effectively reflected the relative retention capacity of seedling traits under Na_2_SO_4_ stress.

Na_2_SO_4_ stress, PH and RL showed a moderate positive correlation (r = 0.50), and GR was also positively correlated with both PH and RL (r= 0.41 for both traits), suggesting partial coordination between seedling growth maintenance and germination performance under stress condition (**Figure 2**). The scatter matrix further illustrates pairwise relationships between traits and showed the discrete distribution pattern of GR-related traits caused by the upper-limit effect of germination (**Supplementary Figure S7**). In contrast, correlation among different STI traits were weak to moderate (**Supplementary Figure S6**), suggesting that PH retention, RL retention, and GR retention may represent partially independent components of seedling-stage Na_2_SO_4_ tolerance.

### 3.5 PCA reveals multidimensional variation in seedling-stage Na_2_SO_4_ tolerance

Principal component analysis (PCA) was performed using the seedling-stage phenotypic traits and salt tolerance indices to summarize the overall structure of salt-stress response among the 134 Xinjiang wheat accessions. The first two principal components explained 60.5% of the total phenotypic variation, with PC1 explaining 44.5% and PC2 explaining 16.1%. The accessions showed clear dispersion in the PCA space, indicating substantial variation in overall seedling performance and Na_2_SO_4_ tolerance within the population (**Supplementary Figure S3**).

Loading analysis showed that traits measured under Na_2_SO_4_ stress and STI-related traits contributed strongly to PC1, suggesting that PC1 mainly reflected salt-stress performance and trait retention capacity. In contrast PC2 was more strongly associated with differences in basal growth under control conditions and selected STI traits (**Supplementary Figure S4**). These results further support that seedling-stage Na_2_SO_4_ tolerance is a multidimensional trait involving multiple phenotypic components rather than a single growth or germination trait (Li et al., 2021; Shan et al., 2022).

### 3.6 Population structure and kinship analysis support the use of mixed-model correction for GWAS

**Figure 3:**
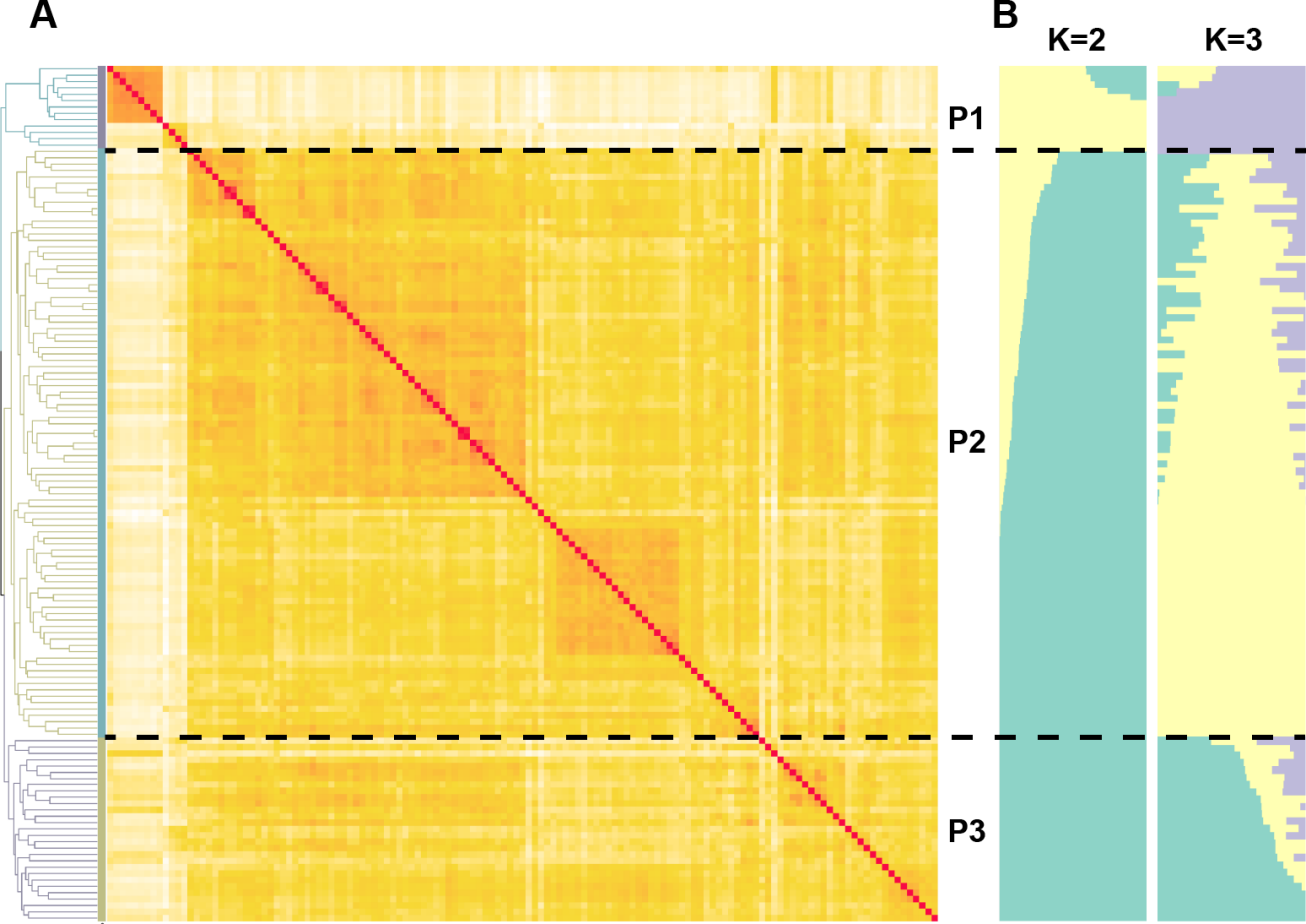
Population structure and genetic relatedness among 134 Xinjiang wheat accessions. **(A)** Heatmap of pairwise genetic distance values calculated as 1 -IBS using LD-pruned genome-wide SNPs together with hierarchical clustering of the accessions. **(B)** Population structure inferred by ADMIXTURE for K = 2 and K = 3. Cross-validation analysis identified K = 3 as the optimal population-structure model. Each vertical bar represents one accession, and the colored segments indicate the estimated ancestry proportion from the inferred genetic groups.

To genetic relatedness and population stratification among the 134 Xinjiang wheat accessions, an IBS-based genetic similarity matrix was constructed using LD-pruned genome-wide SNPs. Pairwise IBS genetic similarity ranged from 0.7660 to 0.9931, with a mean value of 0.8171 and a median value of 0.8166, indicating measurable genetic relatedness among accessions (**Figure 3**). ADMIXTURE analysis was performed for K = 2–3 to K = 10, and the optimal population structure was determined based on cross-validation error. The lowest CV error was observed at K = 3, with a CV error value of 0.60027, indicating that the association panel could be divided into three major genetic groups (**Supplementary Figure S1**). At K = 3, 48 accessions showed evidence of admixture when using a maximum ancestry proportion threshold of 0.8, corresponding to 35.82% of the population. These results confirmed the presence of both population structure and genetic relatedness in the association panel. Therefore, a Linear Mixed Model (LMM) incorporating the genomic relationship matrix was used for GWAS to reduce potential confounding effects caused by population stratification and kinship.

### 3.7 Genome-wide SNP density distribution across wheat chromosomes

Genome-wide SNP density was calculated using 1-Mb sliding windows across the 21 chromosomes of hexaploid wheat. The distribution of SNPs was uneven between chromosomes and different physical regions within individual chromosomes. Overall, some chromosome-arm regions showed relatively high SNP density, whereas regions close to the centromere tended to show lower SNP density (**Supplementary Figure S2**). This pattern is consistent with regional differences in genome structure, recombination frequency, and historical selection pressure. The heterogeneous SNP distribution should be considered when interpreting the uneven distribution of GWAS association signals across the wheat genome.

### 3.8 GWAS identifies loci associated with seedling-stage Na_2_SO_4_ tolerance indices

**Figure 4:**
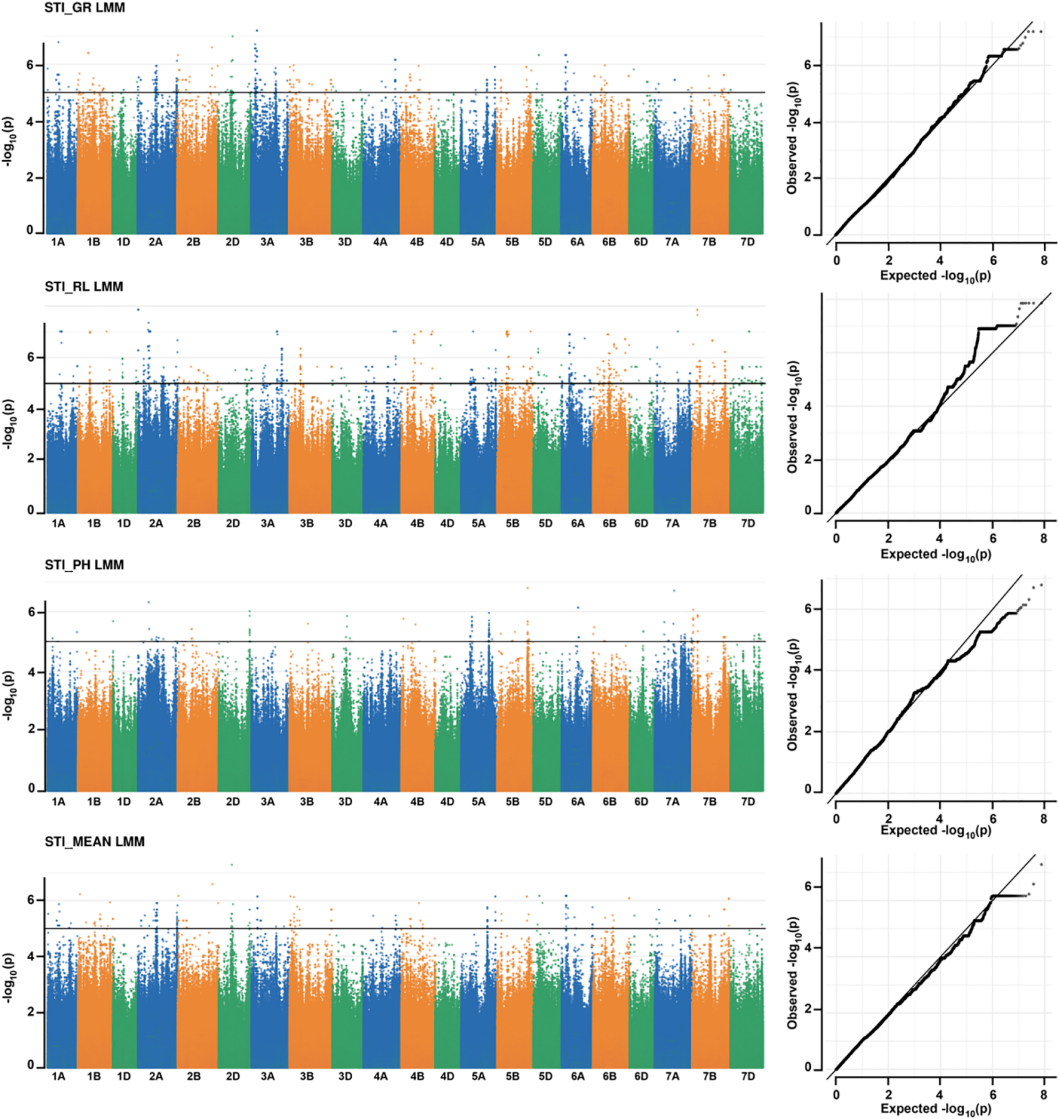
Genome-wide association analysis of seedling-stage salt tolerance indices in 134 Xinjiang wheat accessions. (A-lift) Manhattan plots for STI_GR, STI_RL, STI_PH, and STI_MEAN generated using the Linear Mixed Model (LMM). The horizontal axis representing the physical positions of SNPs across 21 chromosomes of wheat, arranged 1A to7D, and the vertical axis representing −log10(P). The horizontal dashed lines represent the significance threshold of P < 1 × 10^-5^. (B-right) Quantile-quantile plots for the corresponding traits, showing the relationship between observed and expected -log_10_(P) values and allowing assessment of model calibration and potential deviation in the upper tail.

Genome-wide association analysis was performed for STI_PH, STI_RL, STI_GR, and STI-MEAN using a linear Mixed model (LMM) incorporating the genomic relationship matrix to reduce potential confounding effects caused by population structure and genetic relatedness. After SNP quality filtering, association signals with p < 1 × 10–^15^ were retained for further analysis. Multiple significant associations were detected across the wheat genome, and the number and chromosomal distribution of significant SNPs varied among the four salt tolerance indices (**Figures 4A**-left). The Manhattan plot showed distinct association peaks for different STI traits, indicating that PH retention, RL retention, GR retention, and the composite salt tolerance response may be associated with partially different genomic regions. Quantile-quantile plots showed that the observed P values generally followed the expected distribution for most markers, with deviations mainly occurring in the upper tail (**Figures 4B**-right). This pattern indicates that the LMM provided reasonable control of population-related confounding while retaining putative trait-associated signals.

Because the number of significant SNPs can be influenced by trait distribution, phenotyping accuracy, marker density, allele frequency, and the selected statistical threshold, differences in SNP number among traits were not interpreted as direct evidence that any single trait has a more complex genetic architecture. To improve the robustness and biological interpretability of the GWAS results, significant SNPs were subsequently grouped into high-confidence QTL intervals based on the linkage disequilibrium decay distance, as described in section 3.9.

### 3.9 LD-based clustering of significant SNPs identifies 20 high-confidence QTL intervals

**Figure 5:**
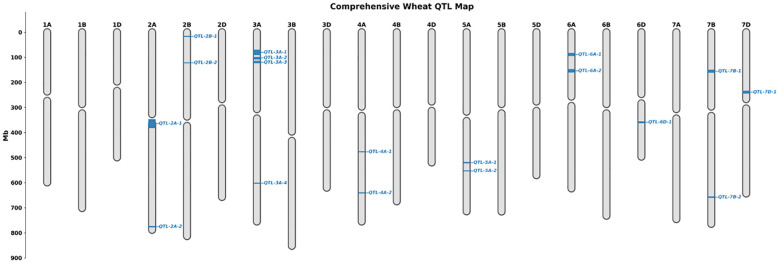
Genome-wide distribution of high-confidence QTL intervals associated with seedling-stage Na_2_SO_4_ tolerance indices. Significant SNPs located within a 1.5-Mb physical window were grouped into the same QTL interval based on the whole-genome LD half-decay distance. Only intervals supported by at least 20 significant SNPs were retained as high-confidence QTLs. The 20 retained QTL intervals were projected onto the 21 chromosomes of hexaploid wheat, showing their chromosomal distribution and multi-trait overlapping regions associated with STI_GR, STI_RL, STI_PH, and STI_MEAN.

To consolidate individual association signals into more robust genomic regions, significant SNPs were clustered according to physical proximity. Based on the whole-genome LD half-decay distance of 1.585 Mb, adjacent significant SNPs located within a 1.5-Mb window were assigned to the same QTL interval. The SNP with smallest P value within each interval was defined as the lead SNP. To improve the reliability of the subsequent candidate-gene analysis, only QTL intervals supported by at least 20 significant SNPs were retained as high-confidence QTLs. Using these criteria, a total of 20 high-confidence QTL Intervals were identified across the four salt tolerance indices (**Figure 5**; **Supplementary Tables S2**–**S7**). Among them, nine QTL were associated with STI_GR, seven with STI_RL, three with STI_MEAN, and one with STI_PH. Several genomic regions contained overlapping signals from more than one trait. For example, the regions on chromosomes 2A at approximately 347.90-380.85 Mb contained overlapping QTL intervals for STI_GR and STI_MEAN. Similarly, overlapping signals for STI_GR and STI_MEAN was detected on chromosome 2B at approximately 15.72-17.25 Mb and on chromosome 6A at approximately 83.85-93.51 Mb.

These multi-trait overlapping regions may contain loci contributing to multiple components of seedling-stage Na_2_SO_4_ tolerance. However, the overlapping association signals do not provide sufficient evidence to confirm pleiotropy, because they may also arise from linkage among neighboring genes or local LD structure. Further validation using haplotype analysis, independent populations, fine mapping, and functional studies is required.

### 3.10 Physiological and RT-qPCR validation of selected salt-tolerant and salt-sensitive accessions

**Figure 6:**
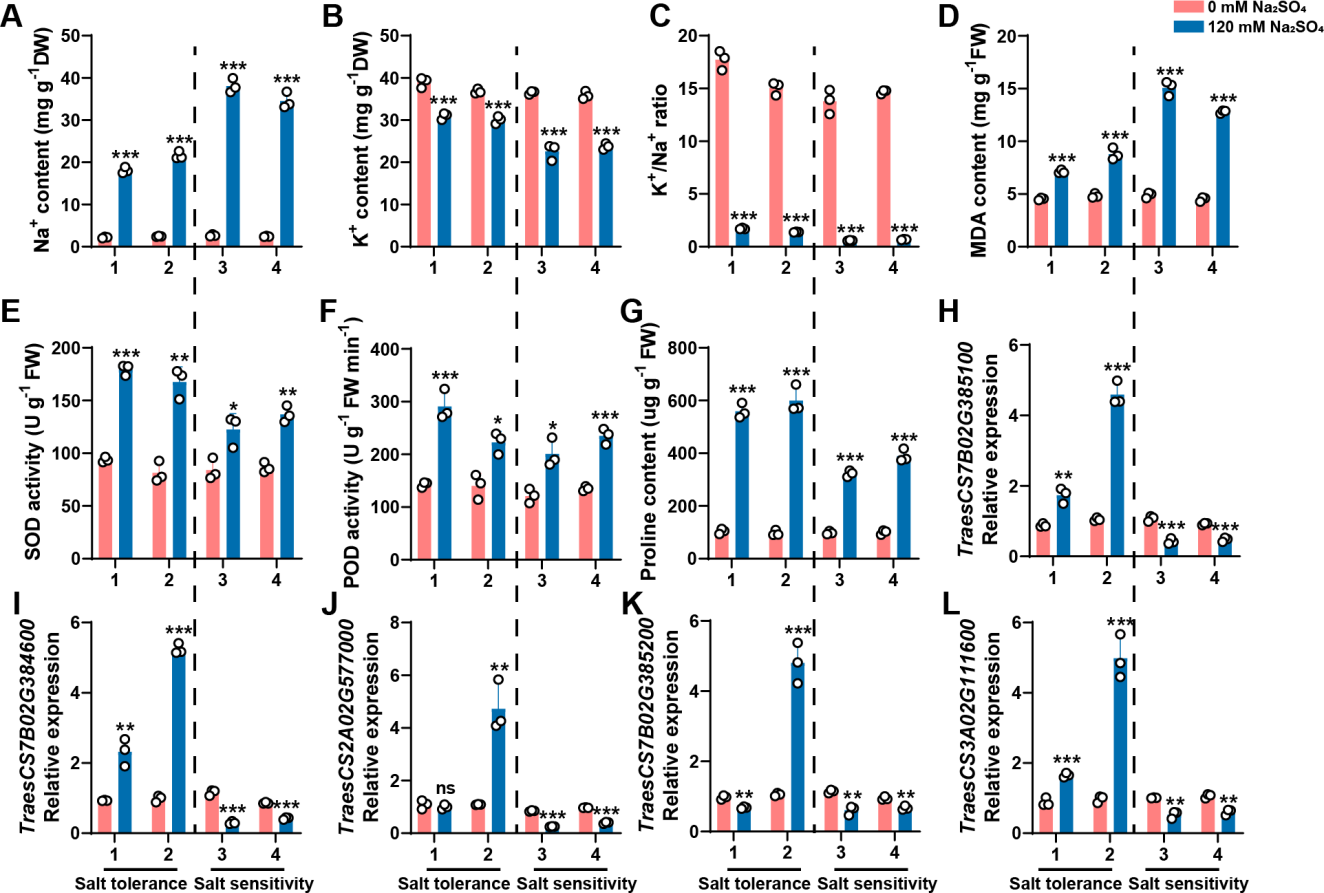
Physiological and RT-qPCR validation of selected salt-tolerant and salt-sensitive wheat accessions under Na_2_SO_4_ stress. Four wheat accessions were evaluated under control conditions (0 mM Na_2_SO_4_) and salt-stress conditions (120 mM Na_2_SO_4_). The first two accessions represent the salt-tolerant group, whereas the last two accessions represent the salt-sensitive group. **(A)** Na^+^ content. **(B)** K^+^ content. **(C)** K^+^/Na^+^ ratio. **(D)** MDA content. **(E)** SOD activity. **(F)** POD activity. **(G)** Proline content. **(H–L)** Relative expression levels of five prioritized candidate genes determined by RT-qPCR. Bars represent mean values, and circles indicate biological replicates. Asterisks indicate significant differences between control and Na_2_SO_4_ treatments: *P < 0.05, **P < 0.01, and ***P < 0.001.

To further validate the salt-tolerance differences between the selected accessions, physiological indicators and candidate-gene expression levels were examined under control conditions and 120 mM Na_2_SO_4_ stress. Compared with the salt-sensitive accessions, the salt-tolerant accessions accumulated less Na^+^, maintained higher K^+^ content and K^+^/Na^+^ ratios, and showed lower MDA content under Na_2_SO_4_ stress. The tolerant accessions also exhibited higher SOD and POD activities and greater proline accumulation, indicating stronger ion-homeostasis maintenance, antioxidant capacity, and osmotic adjustment (**Figures 6A–G**).

The expression patterns of five prioritized candidate genes were further examined by RT-qPCR. These genes showed contrasting responses between the tolerant and sensitive accessions under Na_2_SO_4_ stress, providing additional expression-level support for their prioritization (**Figures 6H–L**).

### 3.11 Transcriptome-supported candidate gene prioritization and RT-qPCR validation

To prioritize candidate-genes within high-confidence QTL intervals, comparative transcriptome analysis was conducted using root tissues collected from first two salt-tolerant accessions (xj1 and xj2) and two salt-sensitive accessions (xj6 and xj7) under control conditions (CK) and 120 mM Na_2_SO_4_ treatment. Each accession-treatment combination included three independent biological replicates, resulting in a total of 24 RNA-seq samples. For comparative analysis the samples were classified into four groups: tolerant accessions under control conditions (Tol_CK), tolerant accessions under Na_2_SO_4_ (Tol_Y), sensitive accessions under control (Sen_CK), and sensitive accessions under Na_2_SO_4_ stress (Sen_Y). The top 30 candidate genes with the highest mean expression levels showed distinct expression patterns among the four RNA-seq groups (**Supplementary Figure S9**). Each group contained six samples, representing two accessions with three biological replicates per accession. To evaluate the reproducibility and overall structure of the RNA-seq dataset, principal component analysis (PCA) was performed using the expression profiles of all 24 root samples. The first two principal components explained 30.57% and 22.58% of the total variance, respectively (**Supplementary Figure S14**). Biological replicates within each material/treatment group clustered closely together, indicating high reproducibility of the RNA-seq data. The PCA clearly separated the eight material–treatment groups and revealed distinct transcriptomic differences among the four wheat accessions under control and 120 mM Na_2_SO_4_ conditions. No obvious outlier samples were detected, supporting the overall quality and reliability of the RNA-seq dataset.

To identify genes showing differential responses between tolerant and sensitive accessions, a formal tolerance group × treatment interaction model was applied. The top 30 candidate genes showing the strongest contrasting expression responses between tolerant and sensitive accessions under Na_2_SO_4_ stress are presented in (**Supplementary Figure S10**). Candidate-gene prioritization was based on the significance of the interaction term, sufficient expression abundance, fold-change magnitude, genomic location within high-confidence QTL intervals, and functional annotation. Five representative candidate genes were selected for detailed visualization. Their expression patterns differed between salt-tolerant and salt-sensitive accessions under control and Na_2_SO_4_-stress conditions (**Supplementary Figure S12**). *TraesCS3A02G111600* and *TraesCS2A02G577000* were induced by Na_2_SO_4_ stress in the tolerant accessions, whereas their responses were weaker or decreased in the sensitive accessions. *TraesCS7B02G384600*, *TraesCS7B02G385105*, and *TraesCS7B02G385200* also showed contrasting expression patterns between the tolerant and sensitive groups. This approach was used instead of relying solely on the numerical difference between expression changes in tolerant and sensitive accessions. The selected genes showed contrasting expression responses between the two accession groups under Na_2_SO_4_ stress, supporting their potential involvement in differential salt-stress responses.

Five prioritized genes were further validated by RT-qPCR: *TraesCS2A02G577000*, *TraesCS3A02G111600*, *TraesCS7B02G384600*, *TraesCS7B02G385100*, and *TraesCS7B02G385200*. These genes were generally upregulated by Na_2_SO_4_ stress in the tolerant accessions but downregulated or more weakly expressed in the sensitive accessions. The RT-qPCR results were strongly consistent with the RNA-seq expression patterns. Across the 20 paired comparisons, the Pearson correlation coefficient between RNA-seq and RT-qPCR log_2_ fold-change values was 0.945 (P = 3.55 × 10^-^¹^0^), and the Spearman correlation coefficient was 0.955 (P = 6.24 × 10^-^¹¹). The direction of expression change was consistent in all 20 comparisons (100%). The RT-qPCR validation results are shown in Supplementary (**Supplementary Figure S2**), and the corresponding RNA-seq and RT-qPCR consistency statistics are provided in **Supplementary Tables S14**–**S16**. These findings provide transcriptome- and RT-qPCR-based support for the prioritized candidate genes. However, further experiments, including haplotype analysis, independent population validation, and functional characterization, are required to determine their precise roles in seedling-stage Na_2_SO_4_ tolerance.

### 3.12 Genotypic-associated phenotypic differences for selected SNPs within prioritized candidate gene regions

To further evaluate the genotypes-phenotype relationship of variants located within the five-candidate gene regions, selected SNPs were examined to their associations with seedling-stage Na_2_SO_4_ tolerance indices. For each candidate-gene region, SNPs reaching the GWAS significance threshold were retained when both homozygous genotype groups (reference allele, 0/0; alternative allele, 1/1) contained at least 10 accessions, thereby reducing the risk of unstable comparisons caused by small group sizes.

For exploratory prioritization, association P values for STI_PH, STI_RL, STI_GR, and STI_MEAN were integrated using Fisher’s method. Within each candidate-gene region, up to four SNPs with the strongest combined statistical evidence were selected for visualization and genotype-group comparison. The selected variants showed significant differences in one or more salt tolerance indices between the two homozygous genotype groups, and several SNPs were also associated with differences in STI_MEAN (**Supplementary Figure S13**).

These results provide additional genotype–phenotype evidence supporting the prioritized candidate-gene regions. However, the observed associations should be interpreted cautiously because they do not demonstrate causal effects of individual SNPs. Variants within the same genomic region may be correlated because of local linkage disequilibrium, and the observed differences may reflect linked polymorphisms rather than the tested SNP itself. Further validation using haplotype analysis, independent populations, and functional experiments is required before these variants can be considered reliable targets for marker development.

## 4 Discussion

This study evaluated seedling-stage Na_2_SO_4_ tolerance in 134 Xinjiang wheat accessions by integrating phenotypic assessment, resequencing-based GWAS, LD-based QTL clustering, and transcriptome-supported candidate gene prioritization. Under control (CK, 0 mM Na_2_SO_4_)) and 120 mM Na_2_SO_4_ stress, plant height, root length, and germination are differed substantially among accessions. Na_2_SO_4_ stress significantly inhibited both seedling growth and germination, while the variation in trait retention among accessions provided a phenotypic basis for identifying genomic regions potentially associated with seedling-stage salt tolerance. Additional physiological measurement in selected tolerant and sensitive accessions further supported the observed differences in salt-stress responses.

Compared with commonly used NaCl treatment, Na_2_SO_4_ stress may impose a partially different ionic environment because it involves both Na^+^ and SO_4_²^-^. In addition to Na^+^-associated ion toxicity and disruption of K^+^/Na^+^ homeostasis, SO_4_²^-^ may influence ionic strength and interactions with nutrient ions (Irakoze et al., 2020; Isayenkov and Maathuis, 2019; Takahashi, 2019). In this study, Na_2_SO_4_ was used as an agronomically relevant stress treatment to evaluate seedling-stage salt tolerance in Xinjiang wheat germplasm. However, because a direct comparison between NaCl and Na_2_SO_4_ treatments was not performed, the identified phenotypes, QTL intervals, and prioritized candidate genes should not be interpreted as evidence of sulfate-specific molecular mechanisms. Furthermore, the osmotic potential and ionic strength of 120 mM Na_2_SO_4_ are not equivalent to those of an equimolar NaCl solution (Reginato et al., 2021; Hadjadj et al., 2023). Future experiments using isotonic or iso-conductivity treatments, together with parallel NaCl and Na_2_SO_4_ conditions, will be required to distinguish general salt-stress responses from ion-specific effects.

The salt tolerance index (STI) demonstrated an interpretable measure of trait retention under Na_2_SO_4_ stress. Each STI was strongly correlated with its corresponding trait under the stress treatment, indicating that the indices captured the ability of accessions to maintain seedling growth and germination relative to the control condition. Correlations among STI_PH, STI_RL, and STI_GR were weaker, suggesting that plant height retention, root length retention, and germination performance represent partially distinct components of seedling-stage salt tolerance (Li et al., 2021). The robustness of the equal-weighted composite index STI_MEAN was further supported by its strong correlations with both the Z-score-based index and the PCA-based index. Root length-related traits showed numerous association signals, which is biologically plausible because roots are directly involved in salt perception, ion uptake, and early stress responses (Khan et al., 2022; Cui et al., 2025; Deja-Muylle et al., 2020). Nevertheless, the number of significant SNPs alone does not demonstrate that root-length retention has a more complex genetic architecture, because SNP counts may also be influenced by trait distribution, marker density, allele frequency, phenotyping precision, and the selected significance threshold.

GWAS and LD-based QTL integration consolidated dispersed significant SNPs into 20 high-confidence QTL intervals. Significant SNPs located within a 1.5-Mb physical window were assigned to the same interval based on the whole-genome LD half-decay distance of 1.585 Mb, and only intervals supported by at least 20 significant SNPs were retained. Several multi-trait overlapping regions were detected, including intervals on chromosomes 2A and 2B. These regions may contain loci contributing to more than one component of seedling-stage Na_2_SO_4_ tolerance. However, overlapping association signals do not provide sufficient evidence to confirm pleiotropy, because they may also arise from linkage among neighboring genes or local LD structure. Further studies using local haplotype analysis, conditional GWAS, independent populations, fine mapping, and functional validation will be required to distinguish single-gene pleiotropy from multi-gene linkage and to assess the stability of these intervals (Liu et al., 2020; Yang et al., 2012; Lu et al., 2020; Chebib and Guillaume, 2021).

The distribution of the high-confidence QTLs was uneven across the three wheat subgenomes. Among the 20 retained QTL intervals, 13 were located in the A subgenome, five in the B subgenome, and two in the D subgenome. The lower number of detected QTLs in the D subgenome may be related to its comparatively lower genetic diversity in hexaploid wheat. Nevertheless, this pattern should be interpreted cautiously because QTL detection can also be influenced by marker density, allele frequency, sample size, and trait-specific statistical power. Several QTLs identified in this study were located on chromosomes previously associated with wheat salt tolerance, including 2A, 2B, 4A, 5A, 6A, 6D, 7B, and 7D. Because previous studies used different populations, salt treatments, genotyping platforms, and reference genome versions, the comparison should be considered at the chromosome or broad physical-region level rather than as evidence of exact locus correspondence. The identified intervals therefore represent priority regions for further validation in Xinjiang wheat germplasm rather than confirmed novel or directly transferable breeding loci.

Transcriptome evidence provides an additional layer of evidence for prioritizing candidate genes within the high-confidence QTL interval. A formal tolerance group × treatment interaction analysis, together with expression-abundance and fold-change criteria, was used to identify genes showing contrasting Na_2_SO_4_ responses between tolerant and sensitive accessions. Five prioritized genes were further validated by RT-qPCR, and the expression changes were strongly consistent with the RNA-seq results, with Pearson r = 0.945, Spearman ρ = 0.955, and 100% agreement in the direction of expression change across the 20 paired comparisons. Physiological measurements also showed that tolerant accessions accumulated less Na^+^, maintained higher K^+^ content and K^+^/Na^+^ ratios, and exhibited lower MDA content but higher SOD and POD activities and proline accumulation than sensitive accessions under Na_2_SO_4_ stress.

Among the prioritized genes, *TraesCS3A02G111600*, annotated as a BBE-like redox-related enzyme, may be associated with ROS homeostasis or cell-wall-related redox processes (Daniel et al., 2017; Costantini et al., 2024). *TraesCS2A02G577000*, encoding a serine/threonine protein kinase may be involved in stress signal transduction and phosphorylation regulation (Lin et al., 2021; Zhang et al., 2023). *TraesCS7B02G384600*, *TraesCS7B02G385100*, and *TraesCS7B02G385200* may be related to cellular structural stability, membrane lipid remodeling, and membrane protection (Le and Ambrose, 2018; Bubier and Schläppi, 2004). Promoter, CDS, and potential transcription-factor-binding-site annotations further support the prioritization of these candidate-gene regions. However, these interpretations remain hypotheses based on integrated association, expression, physiological, and annotation evidence. Additional experiments are required to establish the precise molecular functions of these genes and determine whether the associated variants have causal effects.

Although this study integrated phenotypic, physiological, genomic, and transcriptomic evidence, several limitations remain. First, GR trait values were close to the upper limit under control conditions, producing a ceiling effect that may have reduce sensitivity to genetic differences (Liu and Wang, 2021). Further studies could include more continuous germination related-traits, such as germination potential and germination index and germination speed. Second, root growth was evaluated on filter paper in Petri dishes, which represents a two-dimensional growth environment and does not fully reflect root development in soil. Third, controlled-environment Na_2_SO_4_ stress differs from field conditions in salt composition, water dynamics, temperature, light, and soil interactions. A direct NaCl-versus-Na_2_SO_4_ comparison was also not performed; therefore, the present results should not be interpreted as evidence of sulfate-specific molecular mechanisms. Fourth, the relatively small association panel of 134 accessions limits the power to detect small-effect loci in the complex hexaploid wheat genome. Finally, the candidate genes and associated variants require validation through haplotype analysis, independent populations, field experiments, and functional studies. In addition, transcriptome analysis and RT-qPCR validation were performed only in root tissues, which represent the primary site of salt perception and ion uptake. Future studies should investigate the expression patterns of these candidate genes in additional tissues, such as leaves, to determine whether their responses to Na_2_SO_4_ stress are tissue-specific. Although multiple lines of evidence support the prioritization of these candidate genes, their biological functions remain to be experimentally validated through approaches such as transgenic analysis, gene editing, VIGS, or other functional genomics methods.

Overall, this study revealed significant variation in seedling-stage Na_2_SO_4_ tolerance among Xinjiang wheat accessions and identified high-confidence QTL intervals and prioritized candidate genes regions supported by multiple layers of evidence. These results provide a foundation for future functional validation, marker development, and evaluation of salt-tolerance-associated alleles in independent populations and field environments.

## 5 Conclusion

This study identified substantial variation in seedling-stage Na_2_SO_4_ tolerance among 134 Xinjiang wheat accessions. Treatment with 120 mM Na_2_SO_4_ significantly reduced plant height, root length, and germination rate. Linear Mixed Model-based GWAS and LD-based clustering identified 20 high-confidence QTL intervals, including multi-trait overlapping regions on chromosomes 2A and 2B. Integration of transcriptome analysis, RT-qPCR validation, physiological traits, and variant annotation supported five prioritized candidate genes: *TraesCS2A02G577000*, *TraesCS3A02G111600*, *TraesCS7B02G384600*, *TraesCS7B02G385100*, and *TraesCS7B02G385200*. These findings provide a basis for future functional validation and marker development for wheat salt tolerance.

## Data Availability

The RNA-seq dataset used in this study for candidate-gene prioritization was generated previously and is publicly available in the China National GeneBank Sequence Archive (CNSA). The RNA-seq FASTQ files and LC-MS/MS raw files have been deposited under accession numbers CNP0008138 and CNP0008142, respectively. All additional data supporting the findings of this study are included in the article and its **Supplementary Materials**.
