# Supplementary material for: Phenotypic variation and genetic architecture of seedling salt tolerance in Xinjiang wheat revealed by GWAS and resequencing
Source: Front Plant Sci. 2026 Jul 2;17:1847734. doi: 10.3389/fpls.2026.1847734 (PMC13373106; doi:10.3389/fpls.2026.1847734)
Supplement: Supplementary file 4 [file Table4.docx]

### Supplementary Figures
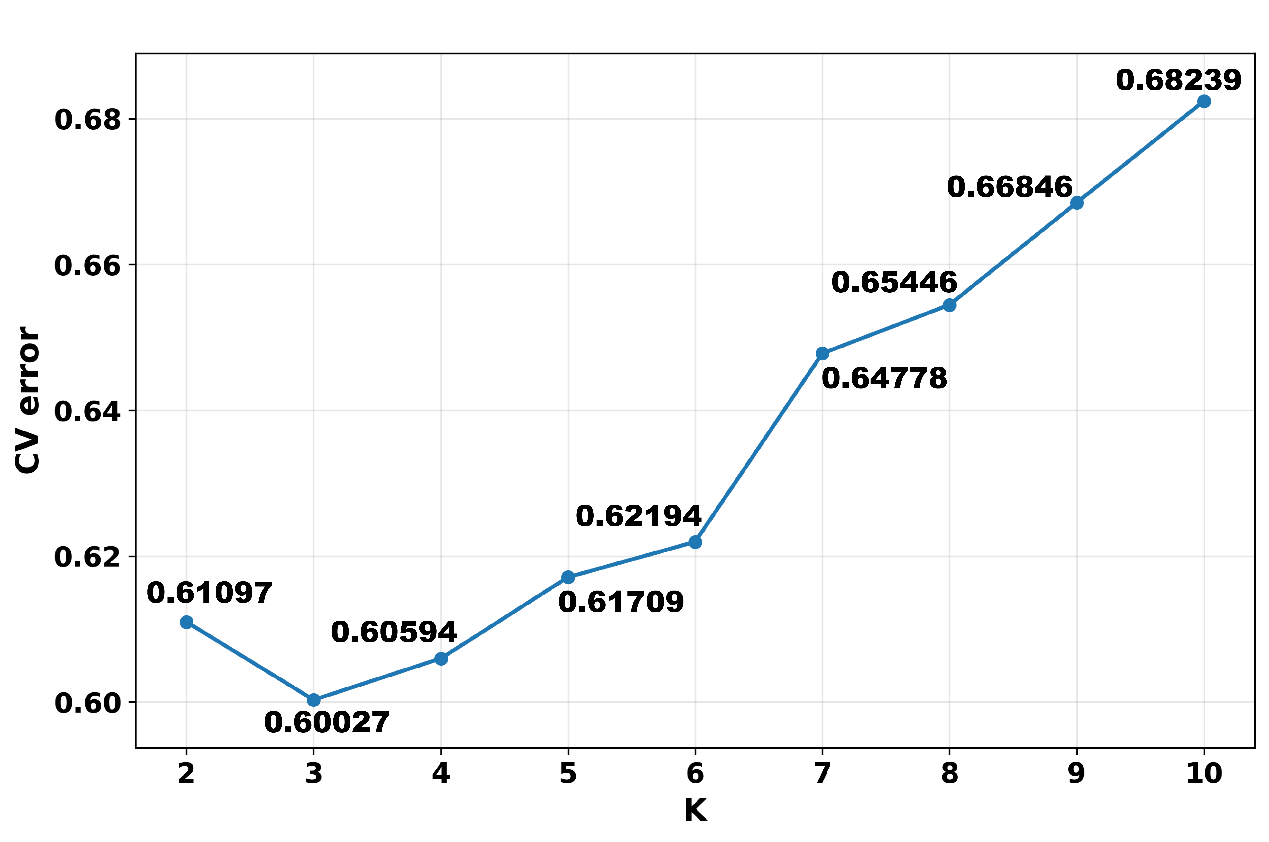


**Fig. S1 Cross-validation error of ADMIXTURE analysis for K values ranging from 2 to 10.** The x-axis represents the assumed number of ancestral populations (K), and the y-axis represents the cross-validation (CV) error. The lowest CV error was observed at K = 3 (CV error = 0.60027), indicating that the 134 Xinjiang wheat accessions were best divided into three genetic subpopulations.

###
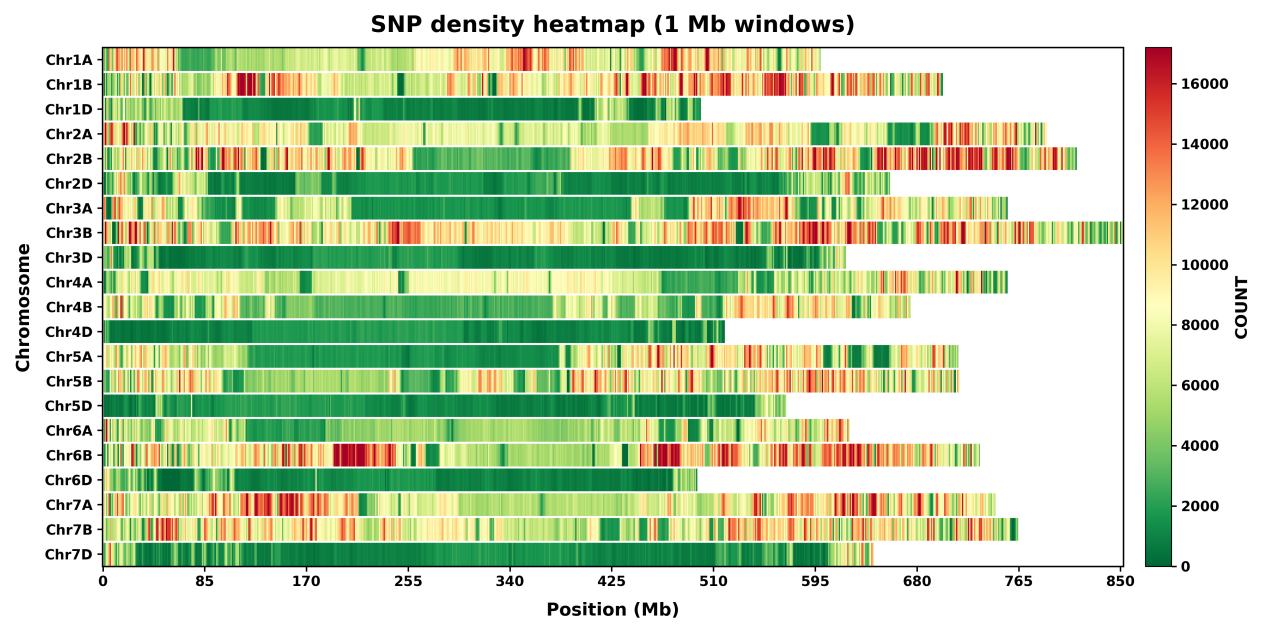


**Fig. S2** Heatmap of SNP density distribution across the whole genome (1 Mb window). The vertical axis represents chromosomes (1A–7D), and the horizontal axis represents physical location (Mb). The color represents the number of SNPs (COUNT) within each 1 Mb window, used to show the spatial distribution differences of variations in the genome.

###
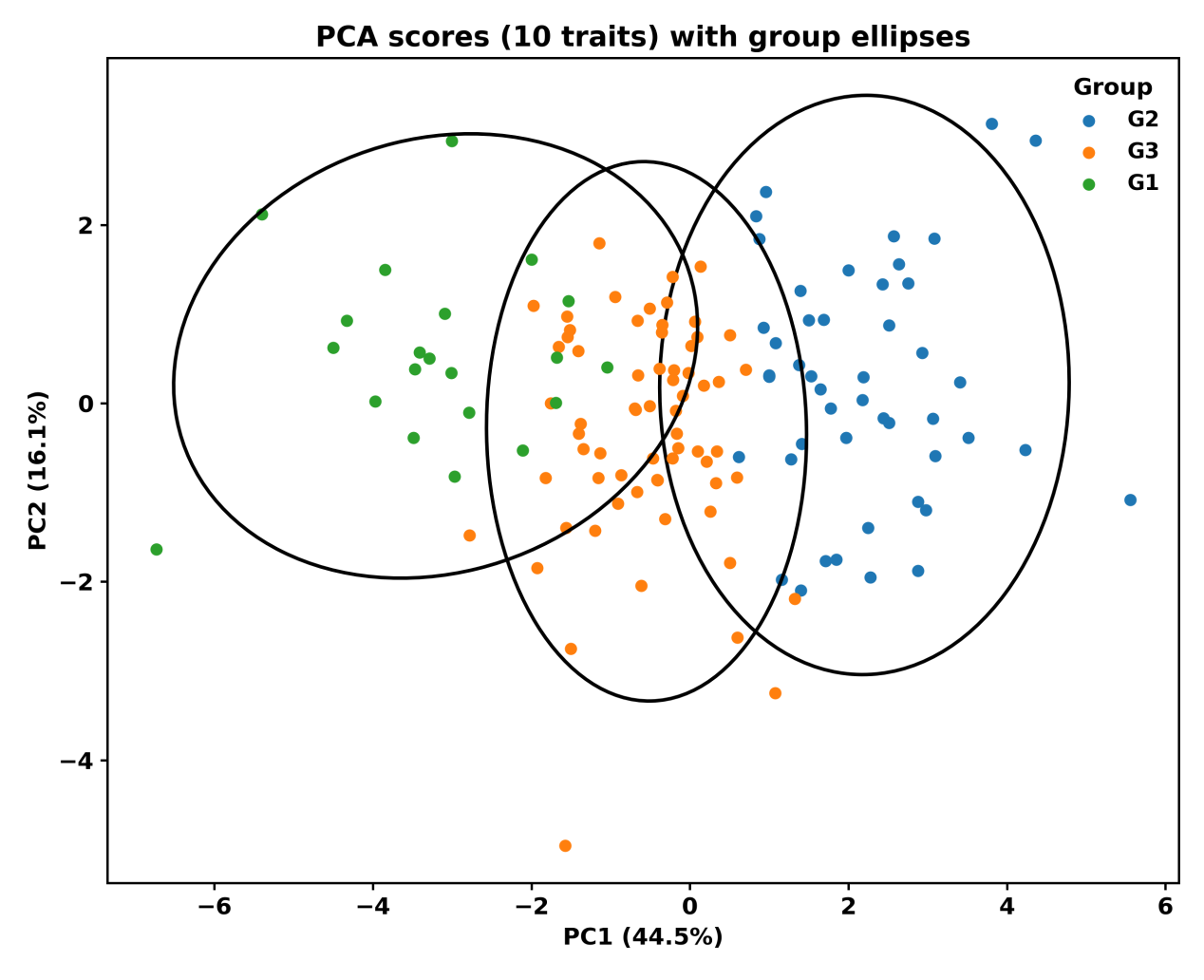
****Fig. S3** Principal component analysis (PCA) score plot of 10 traits. Each point represents a material, different colors indicate groups (G1–G3), and ellipses represent the distribution range within a group; the proportions of variance explained in parentheses for PC1 and PC2 are shown.**

###
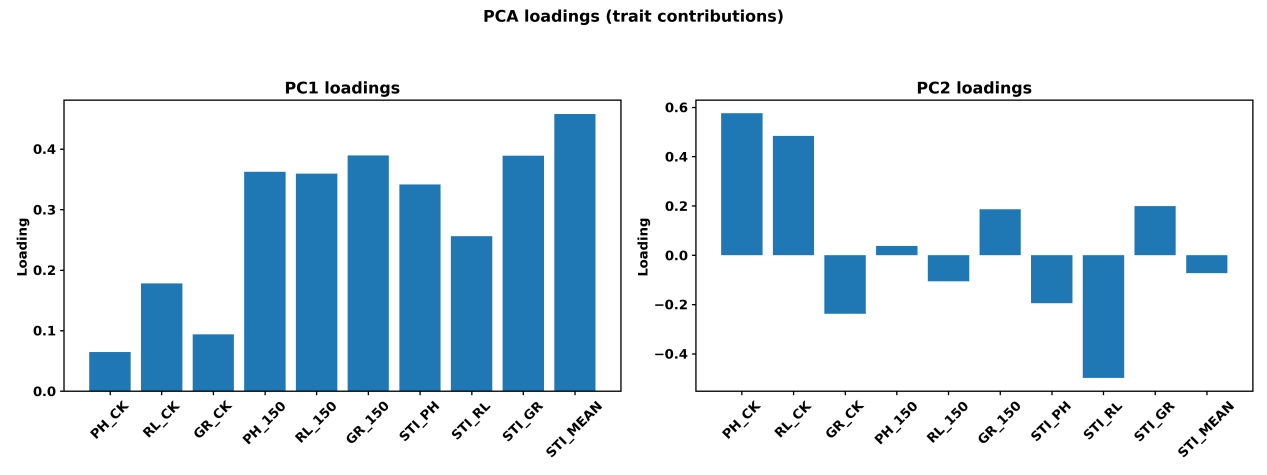
Fig. S4 PCA loading (trait contribution) plot. The left figure shows the loading of each trait in PC1, and the right figure shows the loading of each trait in PC2, reflecting the magnitude and direction of each trait's contribution to the principal components.

###
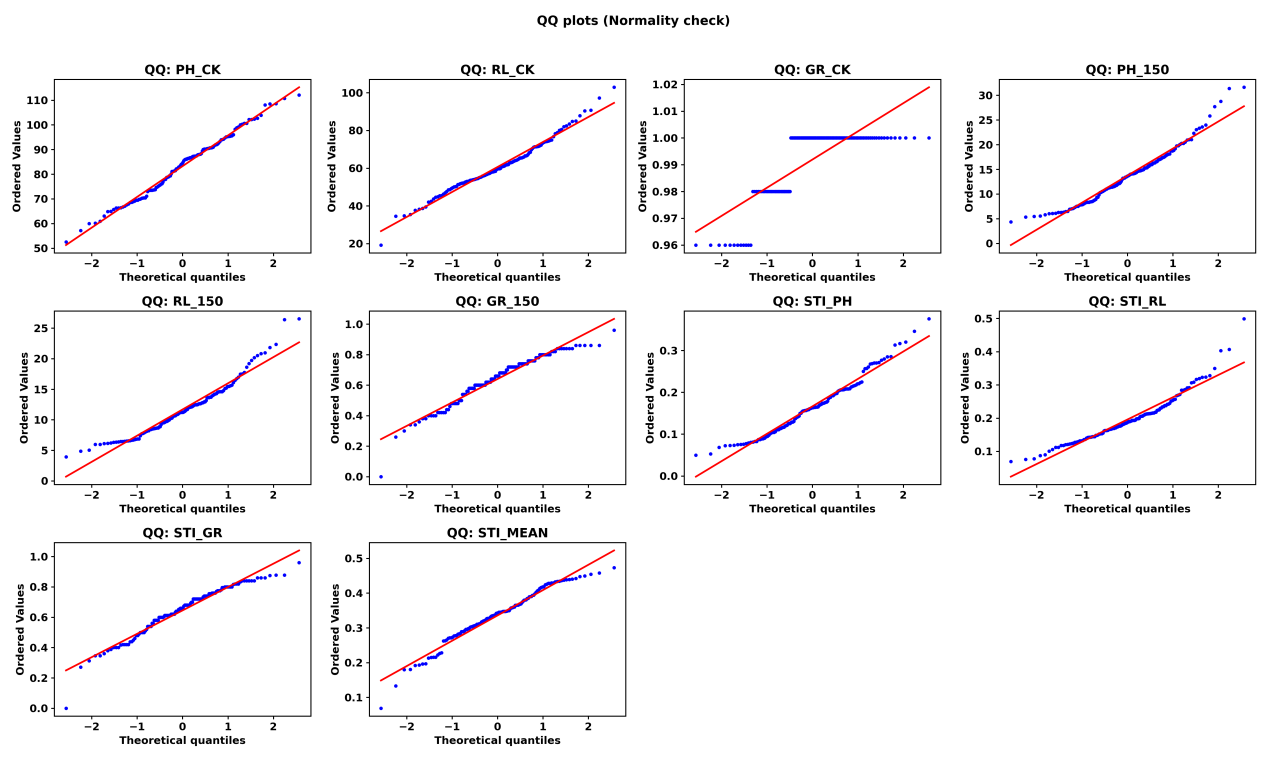


**Fig. S5** QQ plot (normality test) of 10 traits. The horizontal axis is the theoretical quantile, and the vertical axis is the observed quantile; the red line is the theoretical reference line, used to assess the degree of deviation of the phenotypic distribution from the normal distribution.

###
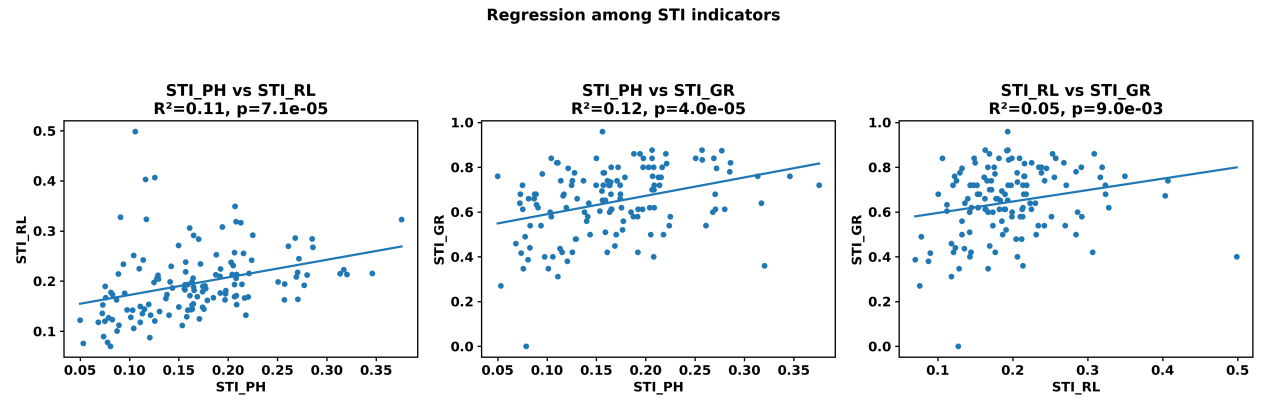


**Fig. S6** shows the regression relationships between STI indices. It displays the linear regression scatter plots and fitted lines for STI_PH vs. STI_RL, STI_PH vs. STI_GR, and STI_RL vs. STI_GR, respectively, with R² and P values ​​given in the figures.

###
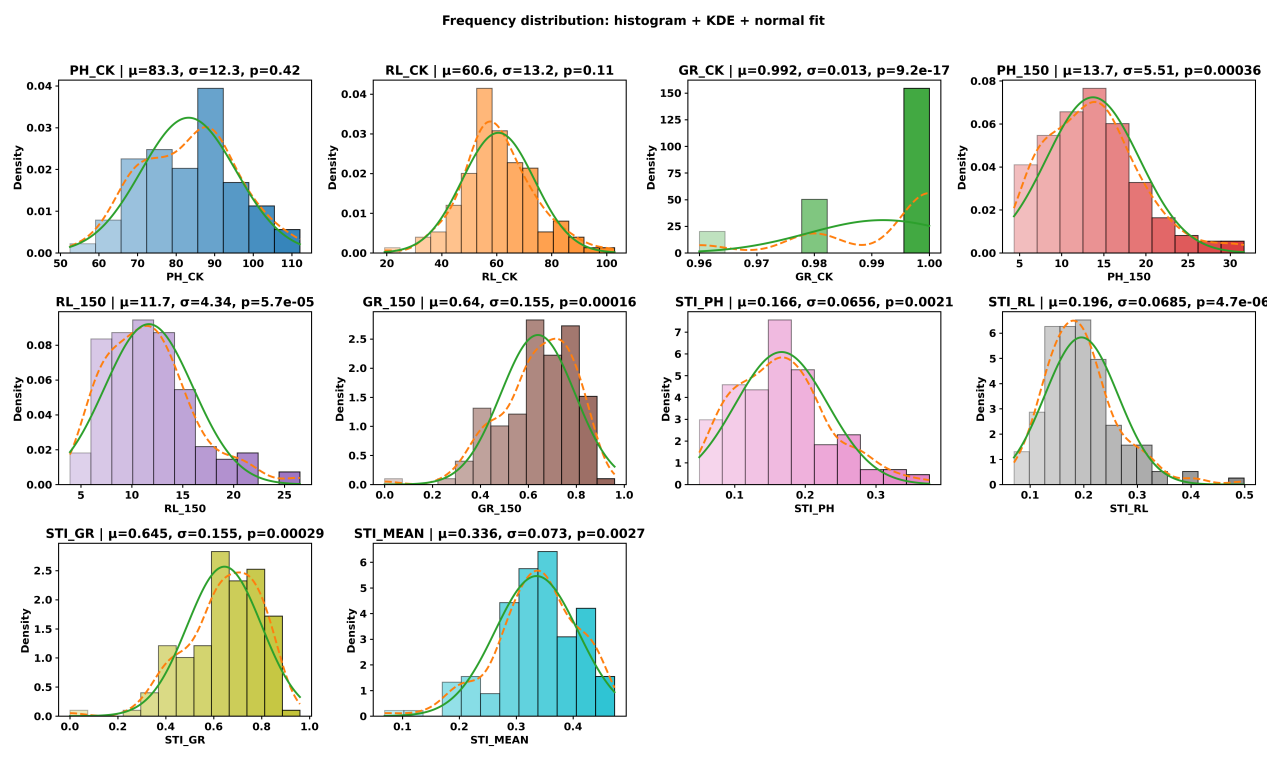


**Fig. S7** Frequency distribution of 10 traits (histogram + kernel density estimation + normal fit). Each subplot shows the histogram, kernel density curve and normal fit curve of the corresponding trait, and gives the mean (μ), standard deviation (σ) and test p-value to describe the trait distribution characteristics.

###
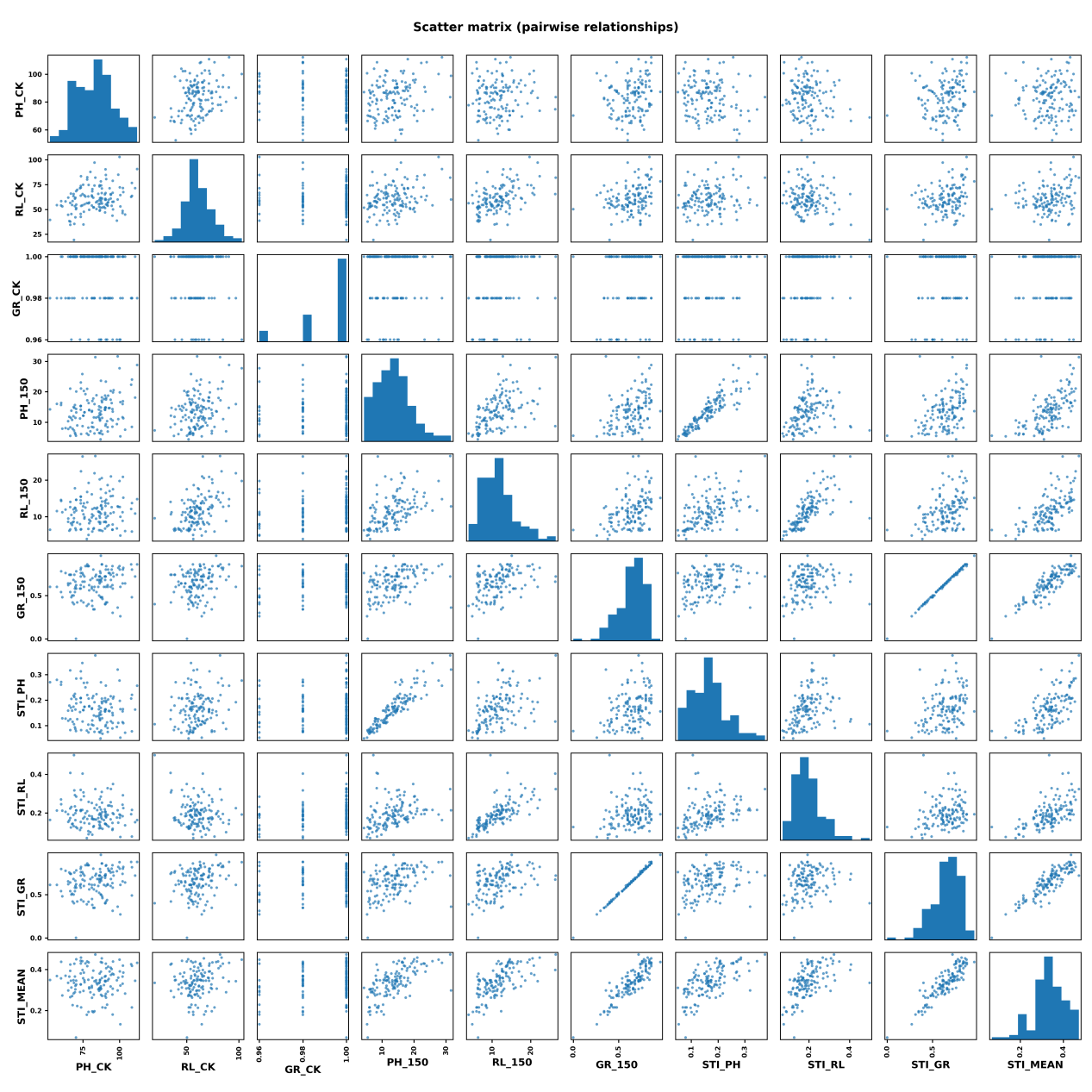


**Fig. S8** shows the scatter plots (pairwise relationships) of the 10 traits. The diagonal lines represent the distribution of each trait (histogram), and the off-diagonal lines represent pairwise scatter plots of the traits, used to show the correlation trends, outliers, and potential nonlinear relationships between traits.

###
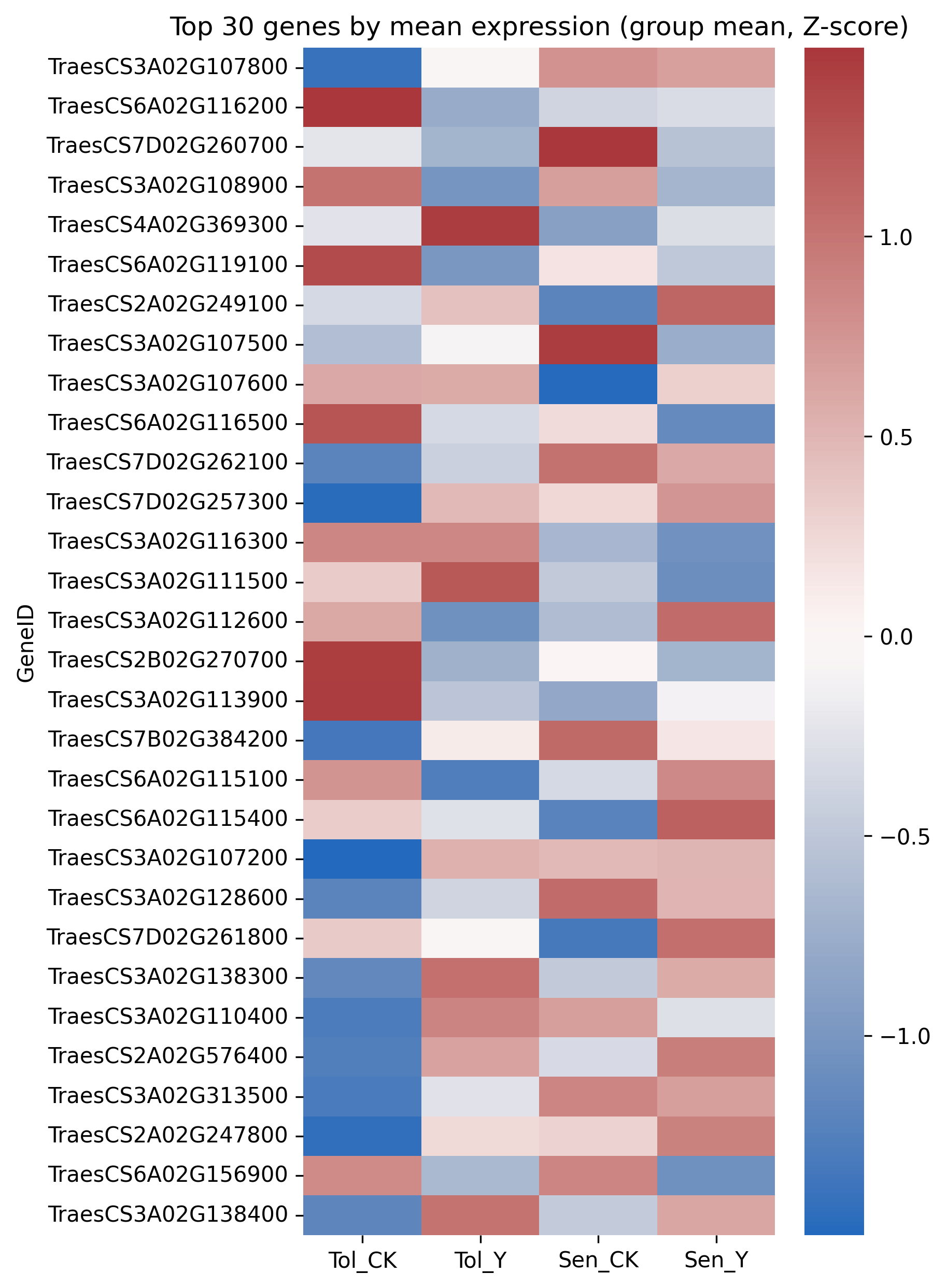


**Fig. S9** shows a heatmap of the expression patterns of the top 30 candidate genes with the highest average expression levels under different material types and treatment conditions. The four columns represent the salt-tolerant control (Tol_CK), salt-tolerant salt treatment (Tol_Y), sensitive control (Sen_CK), and sensitive salt treatment (Sen_Y), with each column showing the average expression level of six samples within the corresponding group. Expression levels were transformed using log2(x+1) and normalized to row-wise Z-scores for each gene. Colors indicate the relative level of the gene among the four groups.

###
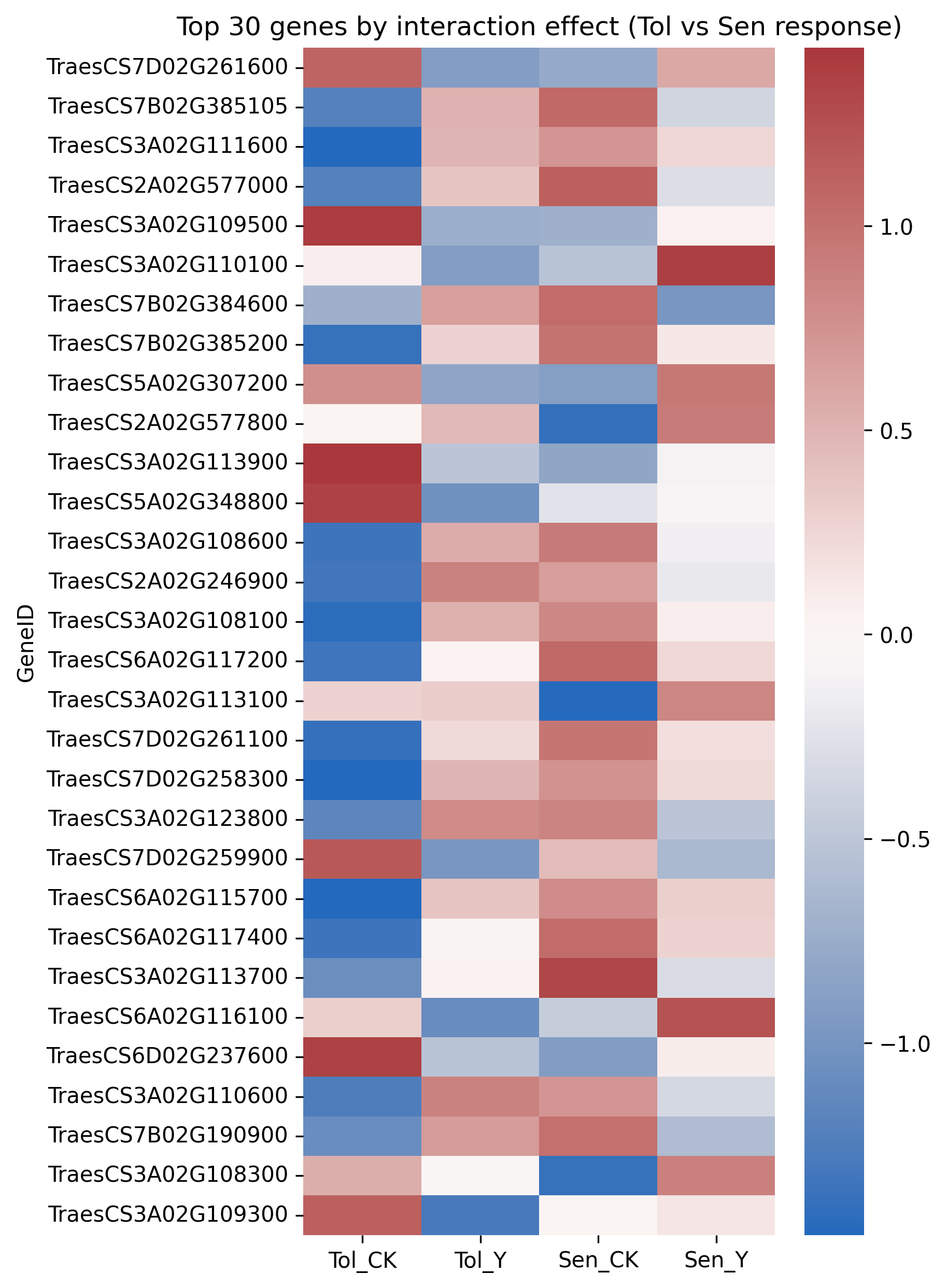


**Fig. S10** shows a heatmap of expression patterns of the top 30 candidate genes screened based on interaction effects. The interaction effect is defined as Interaction = (Tol_Y − Tol_CK) − (Sen_Y − Sen_CK), used to characterize the difference in salt stress response between salt-tolerant and salt-sensitive materials. The four columns are the same as those in Fig. S9, and the colors represent the relative expression levels after Z-score normalization.

###
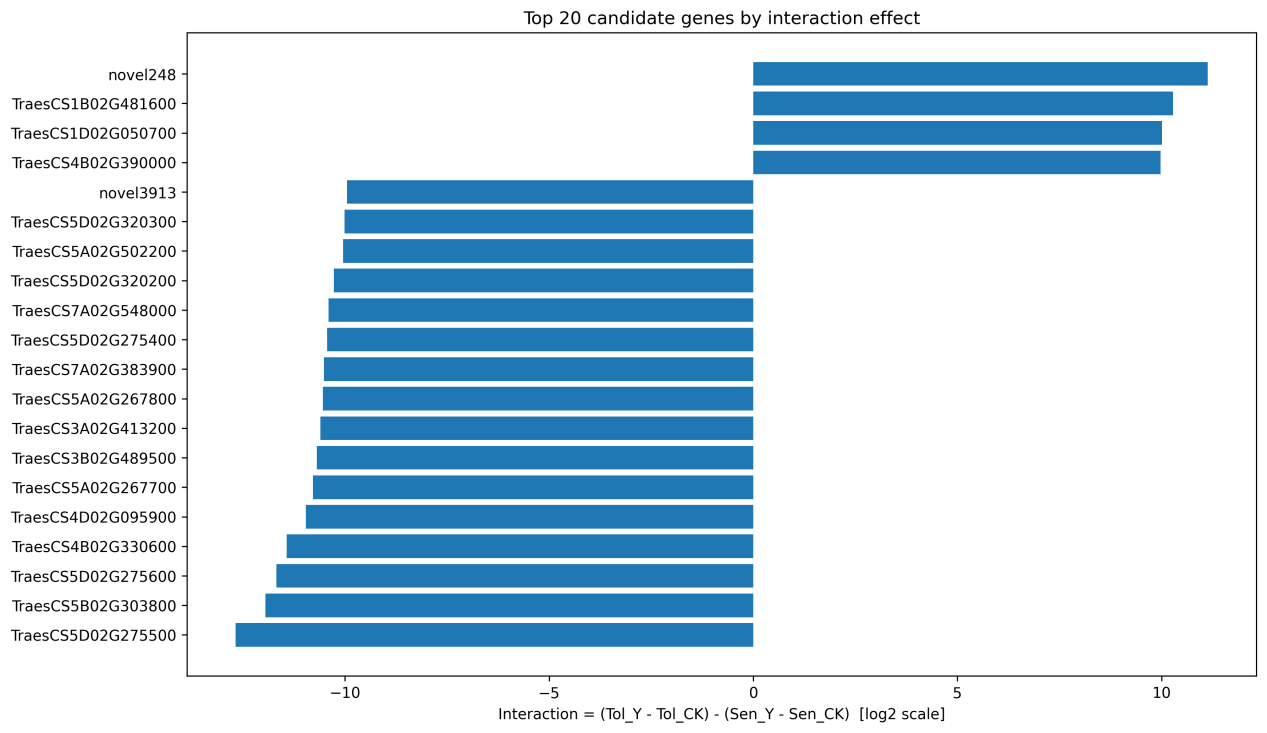


**Fig. S11** shows the top 20 candidate genes with the largest absolute values ​​of interaction effects. The horizontal axis represents the interaction value (log2 scale). Positive values ​​indicate that the salt response amplitude of the salt-tolerant group is greater than that of the sensitive group, while negative values ​​indicate that the sensitive group has a stronger response or that the two groups have opposite response directions.


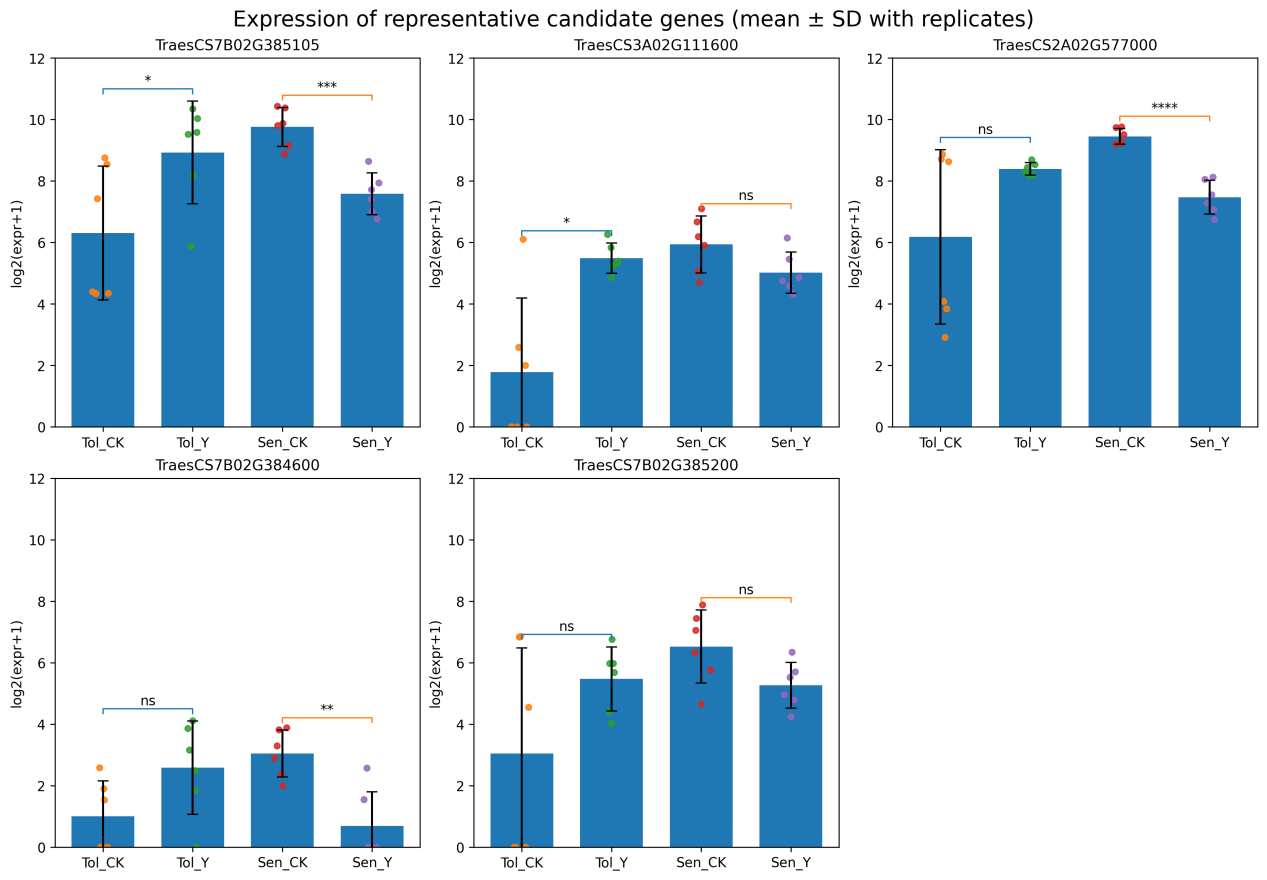


**Fig. S12 shows the expression differences of the top 5 candidate genes with the highest interaction effects in salt-tolerant and sensitive materials. The 24 samples were grouped into four groups: salt-tolerant control (Tol_CK), salt-tolerant salt-treated group (Tol_Y), sensitive control group (Sen_CK), and sensitive salt-treated group (Sen_Y) (n=6 per group). Bars represent group means ± standard deviation (SD), and scatter plots represent replicates for each organism; expression levels were transformed using log2(expr+1). The brackets and asterisks indicate significant differences between CK and salt treatment within the same material type (Welch t-test): ns, not significant; * P<0.05; ** P<0.01; *** P<0.001; **** P<0.0001.**

**
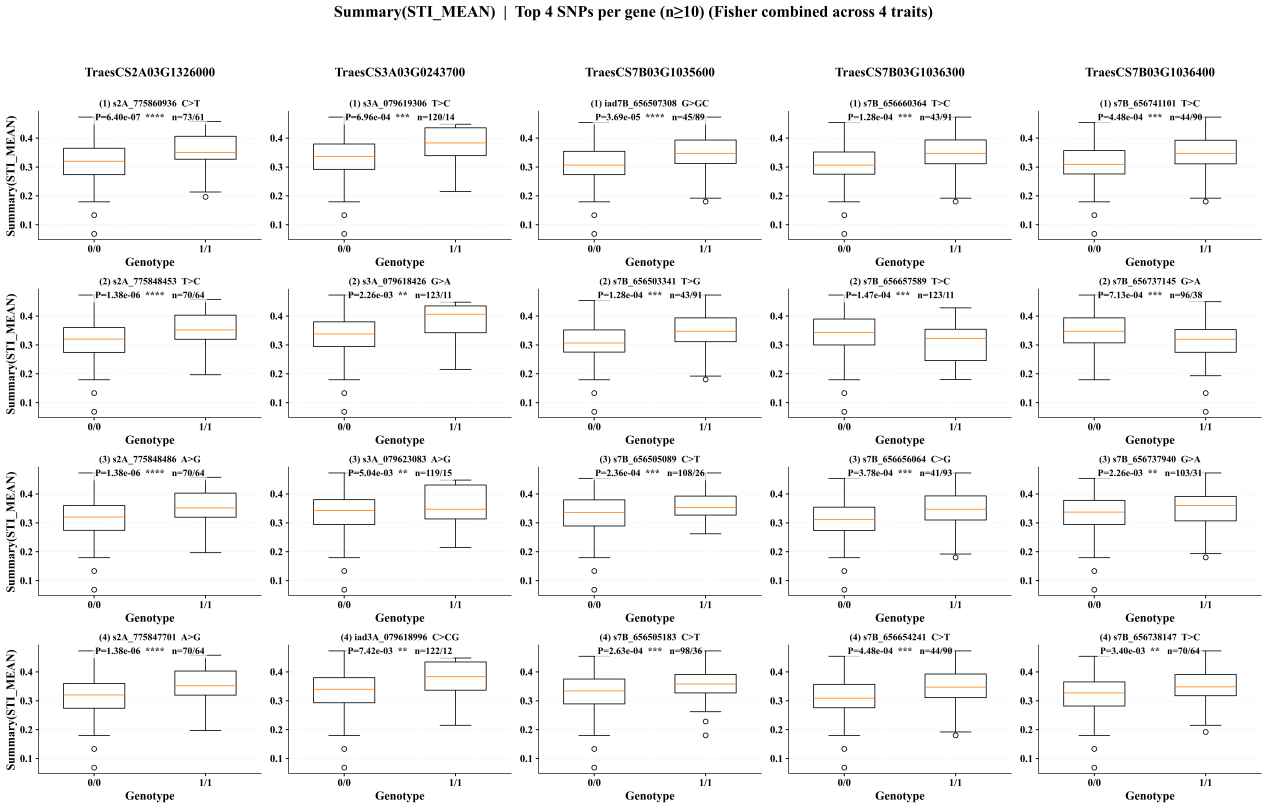
**

**Fig. S13 Genotype effect validation of the top 4 SNPs within five candidate genes (based on Fisher's pooled significance of the four traits). Within the five candidate genes TraesCS2A03G1326000, TraesCS3A03G0243700, TraesCS7B03G1035600, TraesCS7B03G1036300, and TraesCS7B03G1036400, the top 4 SNP loci with the highest overall significance (Fisher's pooled P-value) for each gene and a sample size ≥10 for both homozygous genotypes were selected. Box plots were used to compare the differences in the overall salt tolerance index (STI_MEAN) between materials with different genotypes (0/0 and 1/1); the boxes represent the interquartile range (IQR), the horizontal line inside the box represents the median, the data range must be indicated, and the scatter plots represent outliers. Each subplot is labeled with the Fisher's pooled P-value, significance level, and sample size (n_0/0 / n_1/1). Significance markers: ns, not significant; * P<0.05; ** P<0.01; *** P<0.001; **** P<0.0001.**


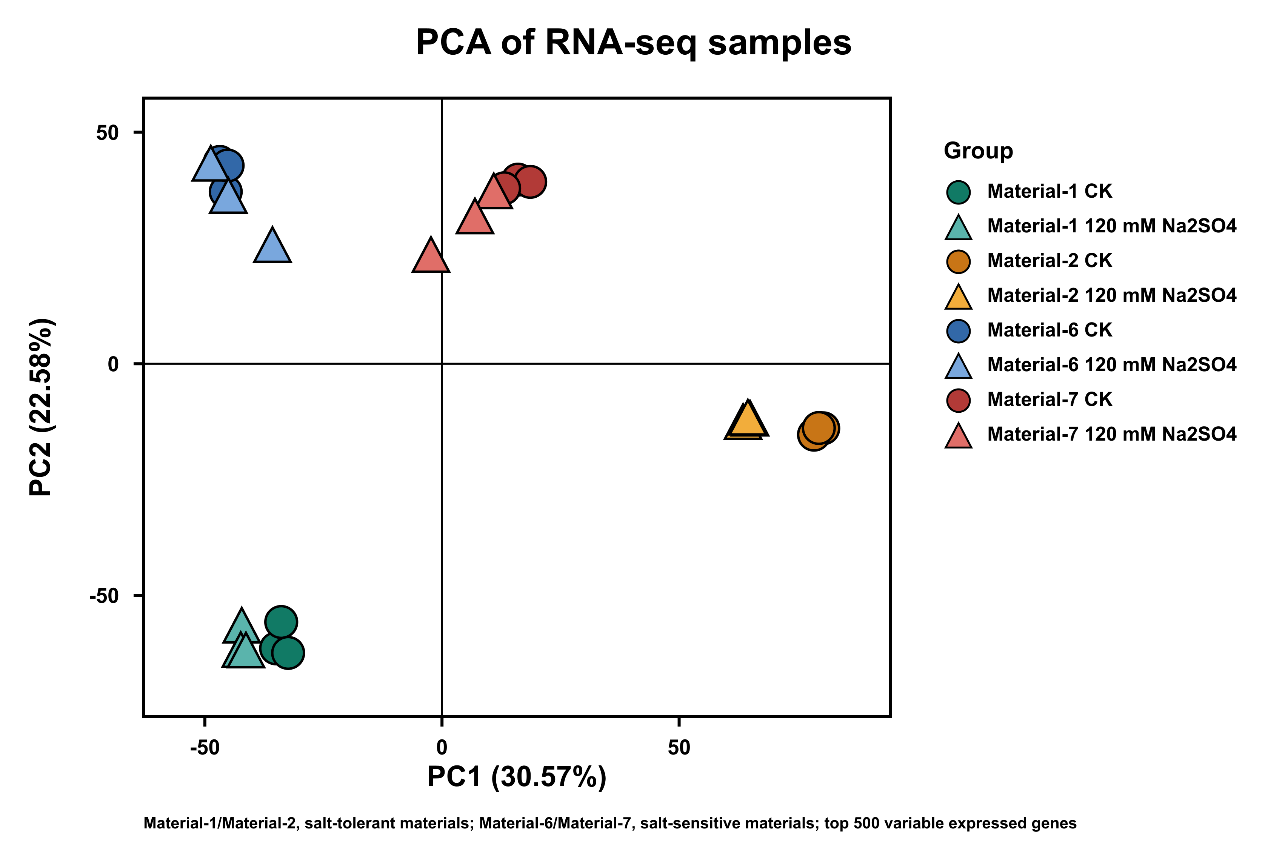


**Figure S14.** Principal component analysis (PCA) of the 24 RNA-seq samples. PCA was performed using the 500 most variable expressed genes from the log₂(expression + 1)-transformed expression matrix. The samples included four wheat materials under control conditions (CK) and 120 mM Na₂SO₄ treatment, with three biological replicates for each material/treatment combination. Material-1 and Material-2 represent salt-tolerant materials, whereas Material-6 and Material-7 represent salt-sensitive materials. PC1 and PC2 explained 30.57% and 22.58% of the total variance, respectively.
